# Adsorption of Cr^6+^ ion using activated *Pisum sativum* peels-triethylenetetramine

**DOI:** 10.1007/s11356-022-21957-6

**Published:** 2022-07-26

**Authors:** Mohamed A. El-Nemr, Uyiosa O. Aigbe, Kingsley E. Ukhurebor, Robert B. Onyancha, Ahmed El Nemr, Safaa Ragab, Otolorin A. Osibote, Mohamed A. Hassaan

**Affiliations:** 1 Department of Chemical Engineering, Faculty of Engineering, Minia University, Minia, Egypt; 2 Department of Mathematics and Physics, Faculty of Applied Sciences, Cape Peninsula University of Technology, Cape Town, South Africa; 3 Department of Physics, Faculty of Science, Edo State University Uzairue, Iyamho, Edo State Nigeria; 4 Department of Technical and Applied Physics, School of Physics and Earth Sciences Technology, Technical University of Kenya, Nairobi, Kenya; 5 Environment Division, National Institute of Oceanography and Fisheries (NIOF), Kayet Bey, Elanfoushy, Alexandria, Egypt

**Keywords:** Aquatic environment, Cr^6+^ removal, Water treatment, Pea peels, Pollution

## Abstract

The adsorption of Cr^6+^ ions from water-soluble solution onto activated pea peels (PPs) embellished with triethylenetetramine (TETA) was studied. The synthesized activated TETA-PP biosorbent was further characterized by SEM together with EDX, FTIR and BET to determine the morphology and elementary composition, functional groups (FGs) present and the biosorbent surface area. The confiscation of Cr^6+^ ions to activated TETA-PP biosorbent was observed to be pH-reliant, with optimum removal noticed at pH 1.6 (99%). Cr^6+^ ion adsorption to activated TETA-PP biosorbent was well defined using the Langmuir (LNR) and the pseudo-second-order (PSO) models, with a determined biosorption capacity of 312.50 mg/g. Also, it was found that the activated TETA-PP biosorbent can be restored up to six regeneration cycles for the sequestration of Cr^6+^ ions in this study. In comparison with other biosorbents, it was found that this biosorbent was a cost-effective and resourceful agro-waste for the Cr^6+^ ion confiscation. The possible mechanism of Cr^6+^ to the biosorbent was by electrostatic attraction following the surface protonation of the activated TETA-PP biosorbent sites.

**Graphical abstract:**

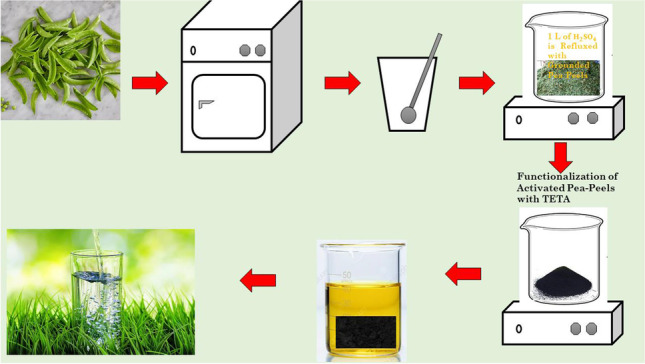

## Introduction

Globally, environmental obliteration issues are presently instigating pollution and impairing natural resources owing to the massive upsurge in the human population and the evolution of industrial activities (El Nemr 2011, 2012a; Ibrahima et al. 2021; Kerry et al. 2021). Continuous progress in scientific and technological advancement is also a contributing factor, and this is now a serious threat to humanity (Eldeeb et al. 2022; Aigbe et al. 2022; Onyancha et al. 2022).

Most of these environmental effluences that are responsible for instigating pollution and impairing natural resources are from industrial wastewater (El Nemr 2005; Aigbe et al. 2020), heavy metals (HMs) (Onyancha et al. 2021a), dyes (Aigbe et al. 2021; Akpomie and Conradie 2021), petroleum spills (El Nemr 2005; Ukhurebor et al. 2021a), gas flare (Onyancha et al. 2021b) and other toxic industrial substances. Reportedly, these increasing industrial environmental effluences are one of the major instigators of the hazardous environmental, health and climatic problems confronting entire ecosystems (the atmospheric, terrestrial and aquatic environments) (Onyancha et al. 2021b; Ukhurebor et al. 2021b). Industrial activities from mining, wood, fertilizer, paper, plating, refining tanneries etc. produce heaps of detrimental noxious effluents containing huge amounts of HMs (Aigbe et al. 2022; Onyancha et al. 2021a) such as chromium (Cr) (Ukhurebor et al. 2021c; Ismael et al. 2020), fluoride (Aigbe et al. 2020) and copper (Cu) (El-Nemr et al. 2022; Medhi et al. 2020), as well as other toxic chemicals that subsequently find their way into the aquatic environment (Ragab et al. 2016; Hosain et al. 2020; El Nemr et al. 2021; Hassaan and El Nemr 2020), and sequentially result in atmospheric, terrestrial and aquatic contaminations. Therefore, eliminating these contaminants is a necessity for safeguarding human health and the environment (Ihsanullah et al. 2106; Aigbe and Osibote 2020; Bilal et al. 2021; Şenol et al. 2021a, 2021b). Recently, biosorbents have gained significant attention as affordable, sustainable materials for the quest for water purification and treatment (Ihsanullah et al. 2106; Aigbe and Osibote 2020; Bilal et al. 2021; Şenol et al. 2021a, 2021b).

However, the emphasis of this current study is on the removal, confiscation or sequestration of Cr^6+^ ions from water using a cheap and eco-friendly technique. Notwithstanding the significance of Cr in several anthropogenic actions, which occurs in several oxidation conditions, with the utmost stable and regularly occurring conditions being Cr^0^, Cr^3+^ and Cr^6+^, the ensuing adulteration in the environment is now a cause of foremost apprehension (Ukhurebor et al. 2021c). Nevertheless, Cr^0^ and Cr^3+^ are dynamic trace elements which at low concentrations of between 0.05 and 1.00 mg/L assist in boosting the development and growth of plants, whereas Cr^6+^ is unessential and harmful to living things (Hassaan et al. 2017a, 2017b; Rowbotham et al. 2020). Hence, it is alleged that Cr is one of the utmost common and controversial elements existing in the mantle of the earth (Ukhurebor et al. 2021c; Vaiopoulou and Gikas 2020).

Reportedly, Cr is typically found in Zimbabwe, South Africa, the Philippines, Kazakhstan, India, Finland and several other nations. They occur as “chromite (FeCr_2_O_4_) in serpentine and ultramafic rocks or multifaceted with some other metallic materials such as bentorite Ca_6_(Cr,Al)_2_(SO_4_)_3_, crocoite (PbCrO_4_), vauquelinite (CuPb_2_CrO_4_PO_4_OH), tarapacaite (K_2_CrO_4_)” etc., and they are extracted in their natural existing form identified as chromate ore (FeCr_2_O_4_) (Xia et al. 2019; Rowbotham et al. 2020; Ukhurebor et al. 2021c). Cr can equally affect the atmospheric environment (air quality) through the manufacturing of coal, and this could eventually lead to adulteration of the terrestrial and aquatic environment. The adulteration of the aquatic environment is discreetly restricted to the surface of the water (Rowbotham et al. 2020).

**Fig. 1:**
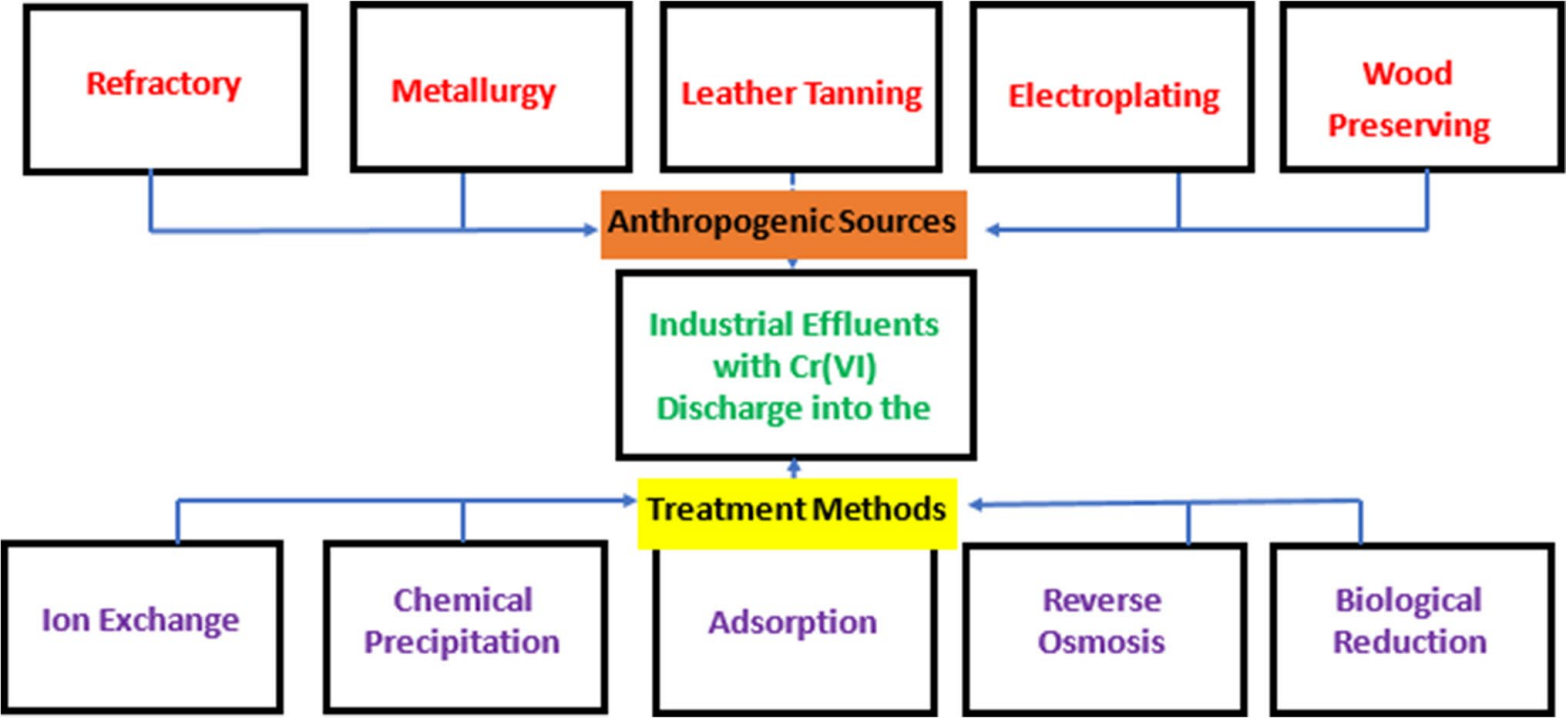
Providence of Cr^6+^ effluence and the existing management measures (Ukhurebor et al. 2021c)

Figure 1 shows some of the topmost human-caused sources of toxic Cr^6+^. The figure also shows the contaminated industrial effluents as well as the management and treatment measures that could alleviate the environmental effluence (Ukhurebor et al. 2021c). The contamination of the aquatic environment with Cr^6+^ occurs possibly as a result of the leakage from the mining of chromate and inapt disposal from mining industries (Cocârţă et al. 2016).

Globally, the need for clean water has risen tremendously (Akpomie and Conradie 2021). The mismanagement and insufficient appropriate treatment techniques for water consistently lead to its adulteration, resulting in its deficiency (Aigbe et al. 2021; Ihsanullah et al. 2016; Aigbe and Osibote 2020; Bilal et al. 2021). However, there are several reported studies on the various techniques for treating and purifying water that is contaminated with Cr^6+^, such as reverse osmosis, ion exchange, biological reduction, chemical precipitation and adsorption (Fig. 1) (Ukhurebor et al. 2021c). To significantly mitigate these challenges of the continuous adulteration of the aquatic environment (water), there is a need to incessantly implement novel, contemporary, cheap and eco-friendly techniques for treating and purifying water. Currently, there are several reported research studies on the utilization of cheap and eco-friendly biosorbents with excellent metal-binding abilities for the confiscation and treatment of contaminated water by HMs (Ismael et al. 2020; El-Nemr et al. 2021; Chen et al. 2018; Villen-Guzman et al. 2019; Ngabura et al. 2018; Duan et al. 2020).

As stated earlier, several agro-materials, such as the peels and leaves of some economical fruits, crops or plants (orange, walnut, mango, banana, sugarcane, watermelon etc.), are now been widely utilized as adsorbents (biosorbent) for the confiscation and treatment of contaminated water by HMs and other noxious chemicals that afterwards find their route into the aquatic ecosystem (Aigbe et al. 2021; Duan et al. 2020; El Nemr 2012b, 2012c). Primarily, the flora cells of these fruits, crops or plants containing cellulose, tannin and lignin have enormous potential to absorb HM ions (El Nemr et al. 2021; El-Nemr et al. 2020a, 2020b, 2020c; Sahlabji et al. 2021).

Consequently, in this study, the possibility of further examining the capabilities of using cheap and natural adsorbents (biosorbents) for the sequestration of Cr^6+^ ions, which are one of the toxic HMs from the water ecosystem, was highlighted. This study is intended to explore the use of a biosorbent decorated with TETA prepared from pea peels (PPs) as a natural biosorbent (activated TETA-PP) for the confiscation of Cr^6+^ ions from the aquatic environment. The batch adsorption studies for the sequestration of Cr^6+^ ions from the water bodies were characterized. Also, the assessment of the optimization of the various parameters/factors using statistical analysis to obtain the best pre-treatment process for the activated TETA-PP that has the maximum adsorption capacity (Singh and Ghatak 2020), as well as the evaluation of the sorption isotherms, kinetics, mechanism of adsorption and regeneration/reusability, was conveniently highlighted.

## Materials and methods

### Materials

The PP (*Pisum sativum*) is an ancient, economical, small sphere-shaped native seed or pod of fruit belonging to the Leguminosae family, mostly common in the northern part of Africa and the western part of Asia as well as other parts of the world. Like every other leguminous plant, PP encompasses symbiotic bacteria known as rhizobia inside the root lumps of their root arrangements. Rhizobia have the distinct capability of fixing nitrogen from molecular nitrogen into ammonia. It comprises a high proportion of consumable proteins, vitamin A and vitamin C, and it is also rich in some essential minerals like calcium and phosphorus (Singh et al. 2020). However, the PP used for this study was acquired from an indigenous market and was utilized for the production of the activated TETA-PP. Sulphuric acid (H_2_SO_4_, *MW* = 98.07 g, 99%) and potassium dichromate (K_2_Cr_2_O_7_, *MW* = 294.19 g, 99 %) and TETA were obtained from Sigma-Aldrich. The standard stock solution of Cr^6+^ ions was prepared from K_2_Cr_2_O_7_. 1,5-Diphenylcarbazide was obtained from BDHZ Chemicals LTD, Poole, UK, as a composite reagent for Cr^6+^ ion analysis. The “Analytik Jena SPEKOL 1300 UV/visible digital spectrophotometer” instrument with matching glass cells of a 1.00-cm optical path, a shaker (“JSOS-500”) and a pH meter (“JENCO 6173”) were used for the experiments and analysis.

### Preparation of biosorbents

PP was appropriately cleaned numerous times with faucet water to remove dust particles and then dried for 2880 min (2 days) at 105 °C. The dried PPs were crushed and grounded. A total of 200.00 g of mashed PP was further boiled for 120 min in 1.00 L of 50% H_2_SO_4_ in a refluxed setting (130 °C), after which it was filtered and washed with pure water until the filtrate became neutral, and then rinsed again with ethanol.

The biosorbent product was dried overnight at 105 °C before being weighed. The processes of sulphonation arose owing to the preparation setting. The produced biosorbent resulting from this reaction was designated as activated PP. The operations of carbonization and sulphonation were carried out during this preparation procedure. TETA was used to functionalize the activated PP, which was then heated in a refluxed setting using a solution of TETA (100.00 mL) for 120 min. The reaction mixture was filtered and rinsed two times with distilled water and ethanol before being used in the next step. The final product was dried overnight at 70.00 °C, and its weight was calculated to be about 36.25 g yield after drying. TETA was included in the product’s label (activated TETA-PP) (El-Nemr et al. 2020a, 2020b, 2020c, 2021).

### Batch adsorption experiment

The stock solution of Cr^6+^ ions (1.00 × 10^3^ mg/L) was made by dissolving 2.83 g of K_2_Cr_2_O_7_ in 1.00 L of distilled water, and this stock solution was further diluted to the predicted concentrations for the calibration standard curve and adsorption tests solution using distilled water. The adsorption capabilities and kinetic features of activated TETA-PP were obtained by the batch sorption studies. A series of “Erlenmeyer flasks (300.00 mL)” was shaken at 200 rpm for a specified time duration with 100.00 mL of the respective concentrations of Cr^6+^ ion solution and varying volumes of activated TETA-PP. With 0.1 M HCl/0.1 M NaOH, the pH of the sample was modified to suitable pH levels.

The pH_PZC_ was studied following the method reported in the literature (Shoaib et al. 2022; El Nemr et al. 2022). The effect of pH on Cr^6+^ ion sorption was observed by mixing 0.1 g of the activated TETA-PP biosorbent with 100 mL of 100.00 mg/L of Cr^6+^ ion solution with changing pH values of 1.48–9.41 using 0.1 M HCl or NaOH solutions. The batch experiment was carried out at ambient temperature (25.00 °C); the suspensions were agitated at 200 rpm for 3 h, and after which samples were taken to measure the equilibrium (EQB) concentration of Cr^6+^ ions.

The biosorbent dosage and contact time (*C*_*t*_) impacts on Cr^6+^ ion removal were examined by shaking of initial Cr^6+^ concentrations of 100.00–400.00 mg/L (100.00 mL) with varying activated TETA-PP biosorbent dosages of 0.20–0.60 g/L at various interval times at 25.00 °C (Şenol and Şimşek 2020).

For the isotherm study, 100.00 mL of Cr^6+^ ion solutions was mixed at 200 rpm for 180 min at 25.00 °C with varying *C*_o_ of Cr^6+^ ion solutions (50.00–300.00 mg/L) and various amounts of activated TETA-PP (0.20–0.60 g/L). The final concentration of Cr^6+^ ions was determined by taking a 0.1-mL sample from the solution in the “Erlenmeyer flask” and separating it from the biosorbent at different time intervals. The concentration of Cr^6+^ ions was quantified using “spectrophotometry and the complexing agent 1,5-diphenylcarbazide (*λ*_max_ = 540.00 nm)” (El Nemr et al. 2007; Eleryan et al. 2019). The EQB adsorption capacities (*q*_e_) were calculated by Eq. (1). Equation (2) was used to estimate the removal % of Cr^6+^ ions from an aqueous solution.

1 $${q}_t=\frac{\left({C}_0-{C}_t\right)}{W}\times V$$

2 $$\mathrm{Removal}\ \left(\%\right)=\frac{\left({C}_0-{C}_t\right)}{C_0}\times 100$$

*q*_*t*_, *C*_o_ (mg/L) and *C*_*t*_ (mg/L) represent the adsorption capacity (mg adsorbate/g biosorbent), which is the biosorbent’s ability to confiscate Cr^6+^ ions from a solution at a certain time; the initial concentration of Cr^6+^ ions; and the residual concentration of the Cr^6+^ ions after the adsorption procedure had been completed for a given time.

### Activated TETA-PP characterization

The “adsorption-desorption (A-D) isotherm” of nitrogen (N_2_) on activated TETA-PP was estimated at the boiling point (BP) of N_2_ gas”. To assess the BET surface area (*S*_BET_) of the biosorbent, a surface area and pore analyser (“BELSORP, Mini II, BEL Japan, Inc.)” using N adsorption at 77 K was utilized (Rouquerol et al. 1999; Gregg and Sing 1982). To estimate the surface area (*S*_BET_) (m^2^/g), monolayer volume (*V*_m_) (cm^3^ (STP)/g), total pore (*p*) volume (*p*/*p*_0_) (cm^3^/g), mean *p* diameter (nm) and energy constant (*C*) for the isotherm, the BET plot was utilized. Equation (3) was employed for computing the average *p* radius.

3 $$r\ \left(\mathrm{nm}\right)=\frac{2{V}_T\ \left(\mathrm{mL}/\mathrm{g}\right)}{a_{s,\mathrm{BET}}\ \left({\mathrm{m}}^2/\mathrm{g}\right)}\times \left(1.00\times {10}^3\right)$$

To estimate the meso*-p* surface area (*S*_mes_), micro*-p* surface area (*S*_mi_), meso*-p* volume (*V*_mes_) and micro*-p* volume (*V*_mi_) of activated TETA-PP, the “Barrett-Joyner-Halenda (BJH) method” and the “BELSORP analysis program software” were employed.

The BJH technique was employed for computing the *p* size distribution (Barrett et al. 1951) for the activated TETA-PP sample. The surface morphology and elemental analysis of the activated TETA-PP sample were explored by utilizing a “scanning electron microscope (SEM) (QUANTA 250)” coupled with an “energy-dispersive X-ray spectrometer (EDX)”. The surface FGs on the activated TETA-PP was investigated employing “Fourier transform infrared (FTIR) spectroscopy (VERTEX 70 linked to Platinum ATR unit model V-100)”. The thermal studies were done employing the “SDT 650 simultaneous thermal analyser device” at a temperature array of 25.00–10.00 × 10^3^ °C, at an increasing rate of about 5.00 °C/min.

### Optimization study using the response surface methodology

To investigate the adsorption of the Cr^6+^ ion using activated TETA-PP, the D-optimal design was assessed using the Stat-Ease Design-Expert v13.0.5.0 program. By employing response surface methodology (RSM), it was possible to optimize the adsorption process’s effective parameters, that is, the effects of three independent variables (*A*, adsorbent dose; *B*, initial Cr^6+^ ion concentration; and *C*, contact time) on the response (*R*, Cr^6+^ ion removal percentage) (RSM). Table 1 outlines the experimental range and variables that will be used.

**Table 1:** The concentration range and levels employed in the batch adsorption investigation using RSM

Independent variables	Notation	Units	Minimum	Maximum	Mean	*SD*
Adsorbent dose	*A X*1	g/L	1	3	2.10	0.8675
Initial concentration	*B X*2	mg/L	100	400	260.00	129.37
Contact time	*C X*3	min	15	180	90.75	65.92

It was determined that six axial points, eight factorial points and six replicates at the central point were necessary to provide the best bespoke design for the three independent variables. By Eq. (4), the number of experiment runs was calculated:

4 $$N={2}^k+2(k)+C={2}^3+2.3+6=20$$

where *N* denotes the number of runs, *k* denotes the number of components to be tested and *C* is the number of experiments carried out at the centre. Table 1 shows the lower and upper bounds of each factor, as well as the median and maximum values. With the help of Stat-Ease Design-Expert v13.0.5.0, we were able to create the experiment data matrix. The generated model was subjected to statistical analysis using the analysis of variance (ANOVA) method. Surface contour plots were used to investigate the interactions that existed between variables.

## Results and discussions

### Activated TETA-PP characterization

**Fig. 2:**
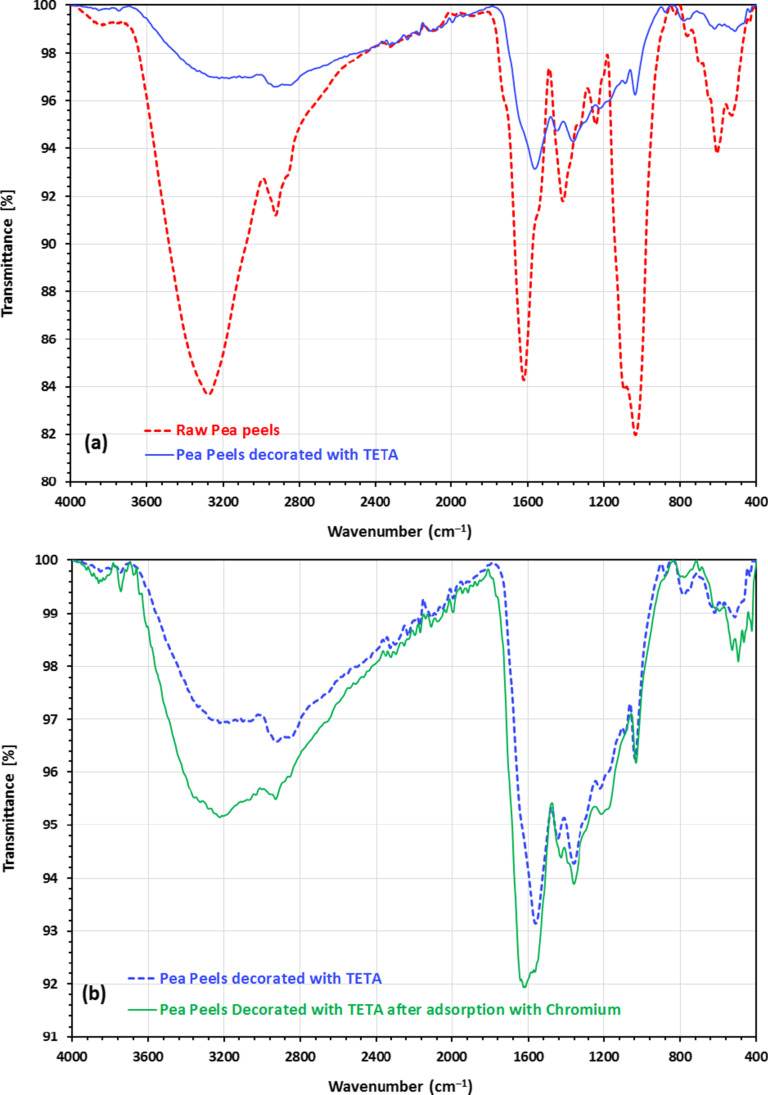
FTIR analysis of **a** raw pea peels and pea peels decorated with TETA and **b** pea peels decorated with TETA and pea peels decorated with TETA after adsorption with chromium

“Fourier transform infrared spectroscopy (FTIR)” was employed for analysing the produced biosorbent to detect the various FGs present. Fig. 2 a shows the raw PP and activated TETA-PP FTIR spectra. Precisely, the strong band at 3223–3377 cm^−1^ agrees with the O–H stretching vibration that occurred in PP, while the wide-ranging adsorption peak around 3170 and 3222 cm^−1^ is indicative of the presence of the –OH FG of glucose and the –NH_2_ of the amino FG in the activated TETA-PP (see Fig. 2a). The occurrence of this new-fangled band indicated that the amino FG was integrated into the biosorbent structure. The –CH stretching vibration that occurred in PP and the activated TETA-PP mixture existed at 2920–2930 cm^−1^. There is no adsorption peak of about 1700 cm^−1^, signifying that the C=O stretching of the carboxyl FG has completely disappeared in activated TETA-PP (see Fig. 2a) (El-Nemr et al. 2020a, 2020b). The existence of the band at 1620 cm^−1^ indicates that the C=O stretching vibration was almost entirely vague in PP and had mostly disappeared in activated TETA-PP.

In the activated TETA-PP, the N–H stretching vibration in fatty amine or aromatic secondary amine was noticed at a frequency of 1572 cm^−1^, signifying that TETA modification may have raised the N–H FG of PP biosorbent-TETA. The adsorption peak at 1408 cm^−1^ specifies that the C–O FG was weak and was found only in PP, while the adsorption peaks at 1437 and 1354 cm^−1^ were ascribed to the stretching vibration of the –N=C=O FG. The presence of this new-fangled peak on activated TETA-PP, which was caused by N-containing FGs, specifies that amino FGs were effectively present following the treatment. When it comes to PP, the existence of oxygenated carbon chains with a peak at 1292 cm^−1^ denotes the C–O–C asymmetric stretching FG, but it is almost completely absent in activated TETA-PP. The bands at 1030–1090 cm^−1^ signify the C–O–H FG that existed in activated TETA-PP. Furthermore, there was an apparent variance between PP and activated TETA-PP peak strength of 1030–1090 cm^−1^, signifying TETA variation on the PP-biosorbent affected the C–O–H FG of activated TETA-PP (see Fig. 2). The effect of chromium adsorption on the peak position is reported in Fig. 2b, and the peaks related to the chromium ion are seen in the wavenumber between 400 and 800 cm^−1^.

**Fig. 3:**
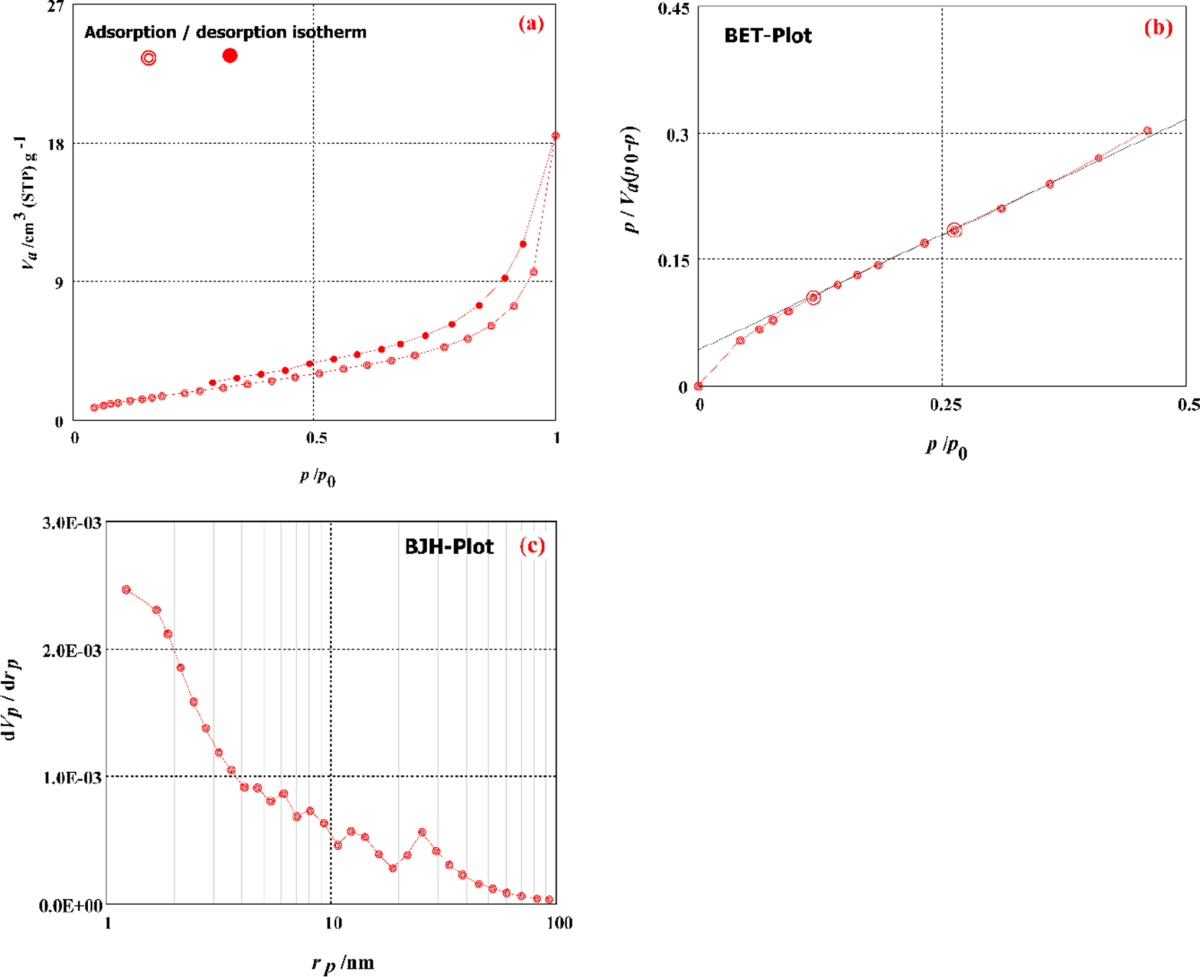
**a** A-D isotherm graph, **b** BET analysis graph and **c** BJH analysis graph of activated TETA-PP

The surface properties of activated TETA-PP were measured by the BET and BJH procedures to give the “BET specific surface area, total *p* volume, mean *p* diameter, monolayer *p* volume, meso*-p* area, meso*-p* volume and meso*-p* distribution peak” and are displayed in Fig. 3. As displayed in Fig. 3, the “BET-specific surface area of activated TETA-PP (7.39 m^2^/g) and the monolayer volume value of activated TETA-PP was 1.70 cm^3^ (STP)/g”. The entire *p* volume value of activated TETA-PP was 0.026 cm^3^/g, and the mean *p* diameter of activated TETA-PP biosorbents was 13.86 nm (meso*-p*). The meso surface area of activated TETA-PP was 8.50 m^2^/g, and the meso*-p* volume value of activated TETA-PP was 0.026 cm^3^/g. The meso*-p* distribution peak value of activated TETA-PP was 1.22 nm. It was described that the *p* in the prepared improved biosorbent might be impeded by the amine FGs (El-Nemr et al. 2020a, 2020b).

**Fig. 4:**
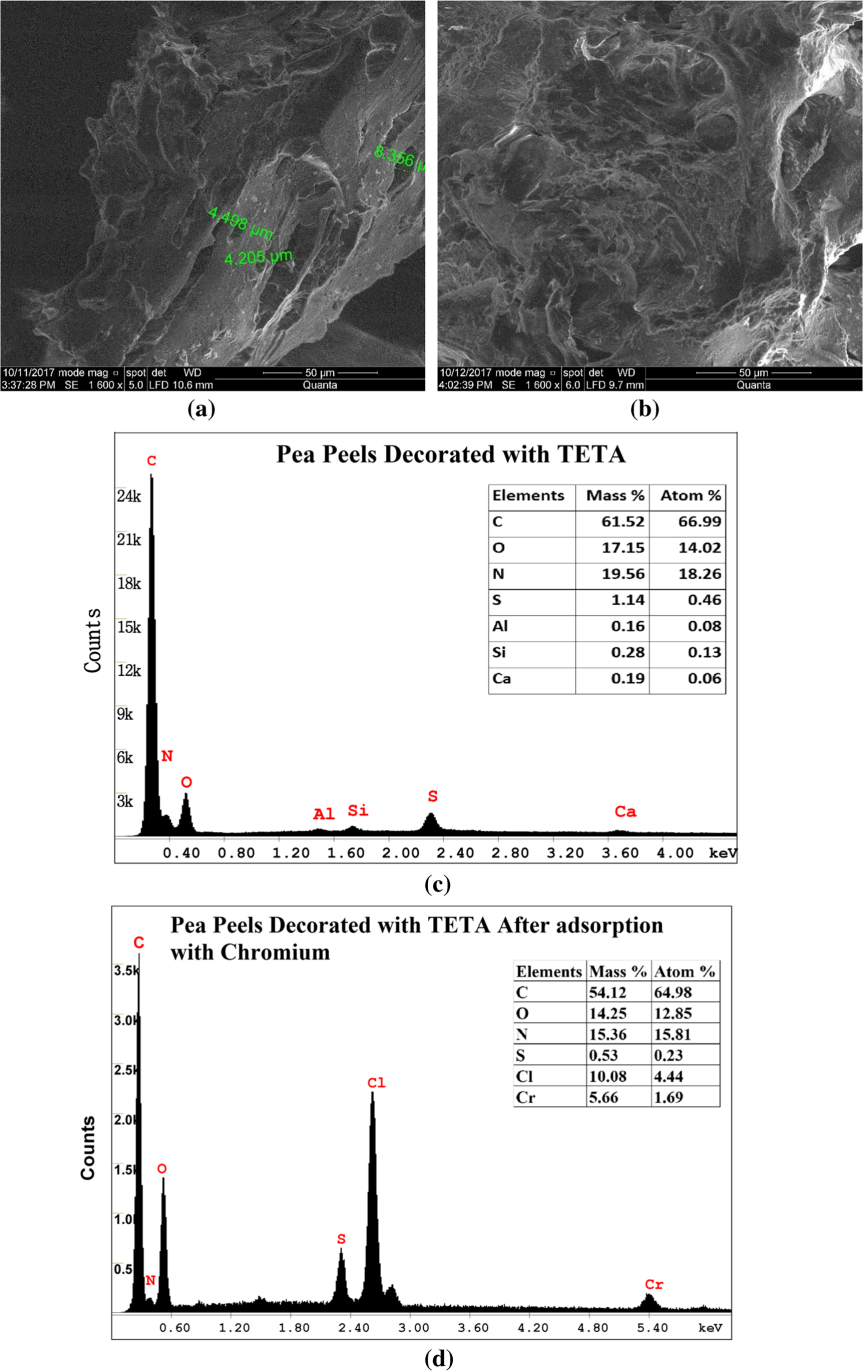
SEM images of **a** PP and **b** activated TETA-PP, **c** EDX analysis of activated TETA-PP prepared from PP and **d** EDX analysis of activated TETA-PP after adsorption with chromium ions

SEM micrographs of the raw PP and the prepared activated TETA-PP were studied (Fig. 4a, b). Fig. 4b displays the surface morphology of activated TETA-PP, and according to the image, some of the *p* and caves had been blocked by the amine creating active sites (El-Nemr et al. 2020a, 2020b). The EDX study was made for the activated TETA-PP for its chemical composition. The composition of chemicals in the activated TETA-PP is described in Fig. 4c. The analysis of the EDX of the activated TETA-PP verified the presence of a 19.56% sample weight for N. The main element in the activated TETA-PP prepared from PP was carbon (61.52%) followed by N (19.56%) and oxygen atoms (17.15%). A minor quantity of sulphur (S) atoms (1.14%) was noted due to the dehydration phase with 50.00% H_2_SO_4_. The composition of chemicals in the activated TETA-PP after being subjected to the adsorption with chromium ions is described in Fig. 4d. The analysis of the EDX of the pea peels decorated with TETA after chromium adsorption verified the presence of a 15.36% sample weight for N. The chromium element in the pea peels decorated with TETA after chromium adsorption was found to be 5.66%, and a new chlorine element was reported as 10.08% due to the pH adjustment with HCl.

**Fig. 5:**
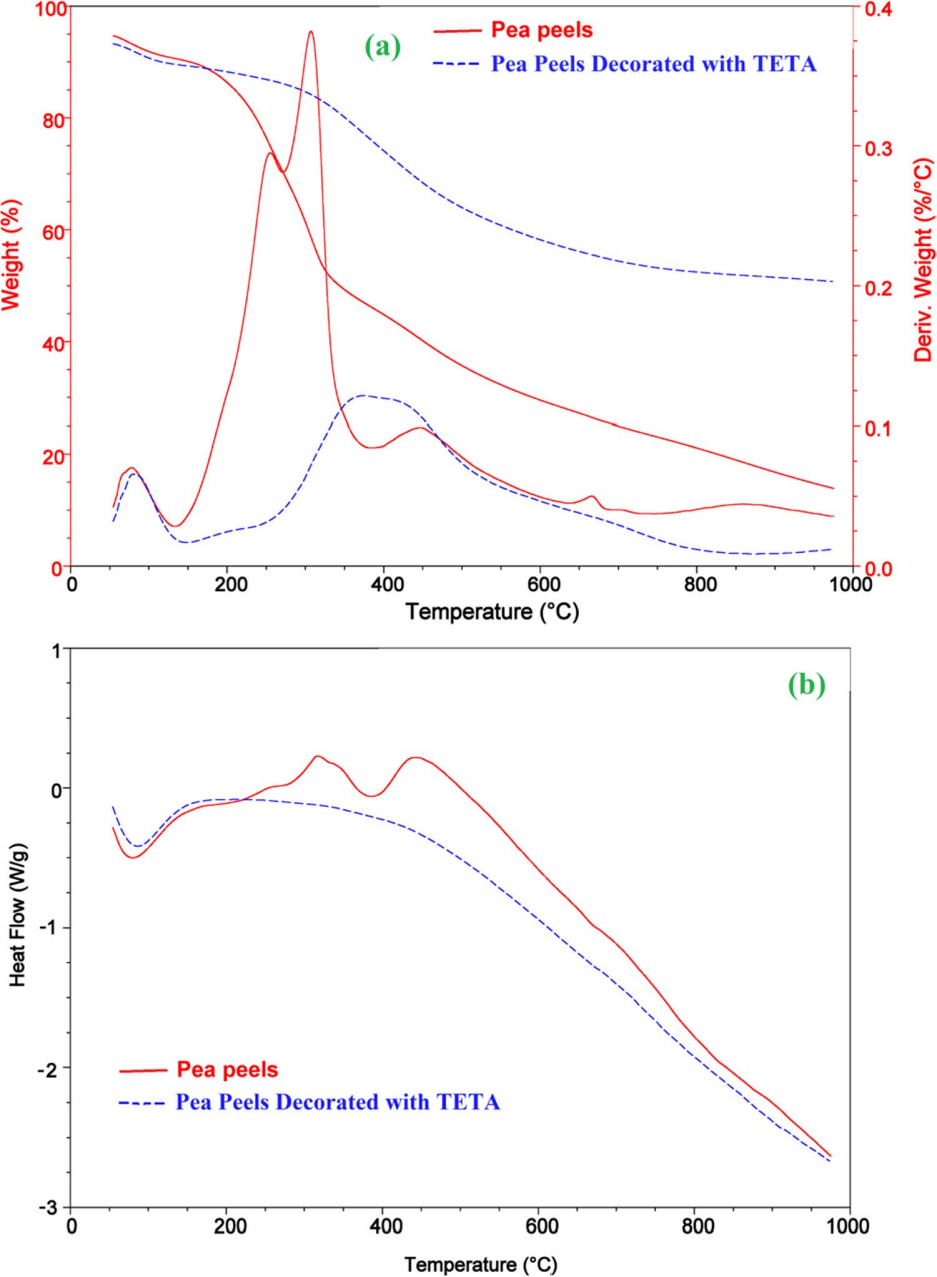
**a** TGA and DTA of the PP and activated TETA-PP and **b** the DSC investigation of the PP and activated TETA-PP

The graphs of thermogravimetry as a function of temperature of the raw materials PP and activated TETA-PP are displayed in Fig. 5. The decomposition of the raw constituent PP ensues in three progressions, while the decomposition of the activated TETA-PP ensues in two phases, as displayed in Fig. 5a. The initial phase occurs at temperatures extending from 50.00 to 150.00 °C, and comprises the loss of surface-bound water and moisture in the sample, with a defined weight loss of 4.05 and 3.95% for raw PP and activated TETA-PP, respectively. The subsequent weight-loss phase had the raw PP losing 63.7% at 150.00–350.00 °C and activated TETA-PP losing 38.50% at 150–1000 °C, respectively. At 350–1000 °C, the raw PP lost about 5.02% of its weight in the third weight-loss stage. The weight of PPs and activated TETA-PP remained constant with percentages of 27.27 and 57.53 obtained, which was indicative of the TETA-modified sample being more stable than the raw PP (El-Nemr et al. 2020a, 2020b).

“Differential thermal analysis (DTA)” could be employed exclusively for classification tenacities. Nevertheless, it is usually utilized for phase diagram (figure or curve) determination, measurements in heat change and decomposition in various settings (Fig. 5a). The DTA figure or curve of the PP sample displays two peaks at a temperature of flow *T*_f_ (76.70 and 342.50 °C). However, the pyrolysis of the PP shows two well-resolved degradation peaks. The DTA of the activated TETA-PP sample displayed mainly comparable two well-determined degradation peaks at a temperature of flow *T*_f_ (83.70, 374.50 °C), and onset points at 56.50 and 260.30 °C. This showed that the steadiness of the activated TETA-PP sample improved by decoration with TETA.

Thermal transitions could be employed in comparing constituents using “differential scanning calorimetry (DSC)”. The DSC of the PP and activated TETA-PP is displayed in Fig. 5b. All of the samples had crystallization temperatures *T*_C_ below 100.00 °C, which could be ascribed to the crystallization of the water molecules. The crystallization temperature *T*_C_ of raw PP was found to be between 348.30 and 758.60 °C utilizing the DSC. The other phase transitions are not visible in the DSC of activated TETA-PP (El-Nemr et al. 2020a, 2020b).

### Impact of different parameters on Cr^6+^ ion sorption

A key parameter in a sorption medium that controls the adsorption of HM ions to the active sites on the adsorbent surface is pH. The initial pH of the adsorption medium is a crucial element in describing the sorption mechanism and what impacts the ionization of the sorptive molecular particles as well as the sorbent surface charge. The chemical interaction and electrostatic force which corresponds to the Columbic force (repulsion or attraction as the case may be) between re-active sites and sorbing ions are the two main factors that are necessary for the adsorption process (Aigbe et al. 2018a).

**Fig. 6:**
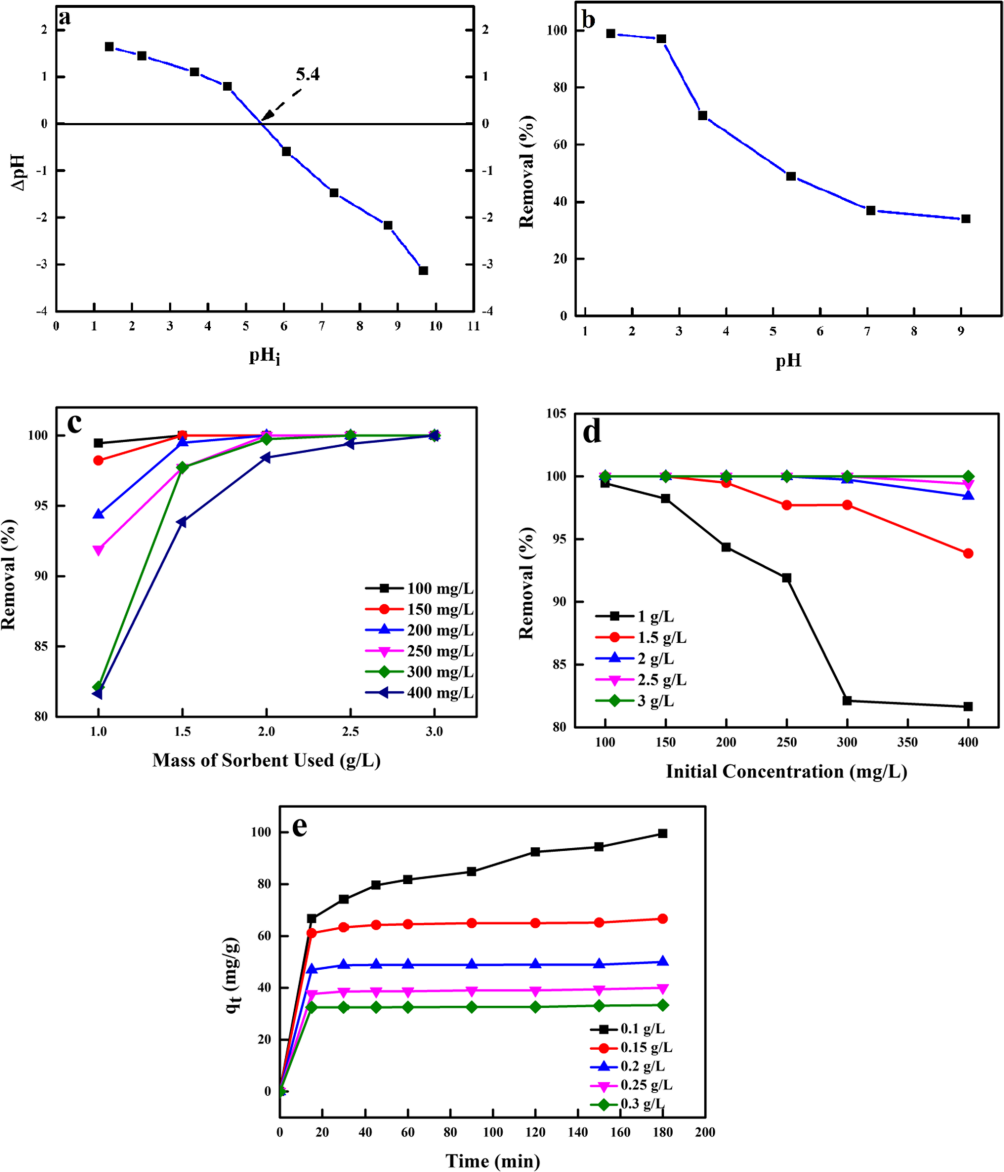
**a** pH_PZC_ of the activated TETA-PP, **b** the impact of pH on the adsorption of Cr^6+^ ions from water by activated TETA-PP, **c** impact of dosage of activated TETA-PP used for Cr^6+^ ion removal, **d** impact of beginning Cr^6+^ ion concentrations on the adsorption efficiency using activated TETA-PP biosorbent and **e** impact of *C*_*t*_ on Cr^6+^ ion sequestration to biosorbent.

From the result shown in Fig. 6a, the point of zero charge (pH_PZC_) was estimated to be 5.4. When the pH of the solution was less than the pH_PZC_, the active sites on the biosorbent surface were positively charged, and when the pH of the solution was greater than the pH_PZC_, the active sites on the biosorbent surface were negatively charged. As observed in Fig. 6b, the percentage (%) of Cr^6+^ ions removed onto the biosorbent decreased with the increasing pH of the solution (99–34%), with optimum % removal noticed at pH 1.6 (99%). Cr^6+^ is reported to exist in different species at various pH values such as H_2_CrO_4_, HCrO_4_^−^, CrO_4_^2−^, HCr_2_O^7−^ and Cr_2_O_7_^2−^ (Aigbe et al. 2020). At pH 1–6, the dominant species of anionic chromium (Cr) were H_2_CrO_4_, Cr_2_O_7_^2−^ and HCrO_4_^−^ (in acidic solution when pH ≤ 2). The determined sorption of Cr^6+^ ions onto the biosorbent was noticed at pH 1.6, which was ascribed to the electrostatic force (attraction) between the positively charged active sites on the biosorbent and the negatively charge monovalent chromate ion (Cr_2_O_7_^2−^). As the pH of the solution was amplified (pH > 6.5), Cr^6+^ occurs in the form of CrO_4_^2−^. This led to increased competition between negatively charged OH^−^ ion and CrO_4_^2−^ specie for the active sites on the sorbent surface, thereby leading to the electrostatic force (repulsion) between negatively charged sites on the biosorbent and the Cr specie (CrO_4_^2−^), thereby causing a reduction in the % of Cr^6+^ ions eliminated (Aigbe et al. 2018a; Anah and Astrini 2017). Depending on the pH of the water-soluble solution and under oxygenated conditions, Cr^6+^ can exist as HCrO_4_^−^ and CrO_4_^2−^. These oxyanions are easily decreased to a trivalent (Cr(III)) state when the electron donors (reducing reagents) are present, and these reduction rates improve as the pH is decreased (Chang 2005). It is assumed that Cr^6+^ is sorbed onto the surface of the activated TETA-PP and remains attached to the activated TETA-PP via electrostatic attraction, while other parts receive electrons from nearby electron carriers such as O and N groups in the biosorbent, leading to the subsequent reduction of Cr^6+^ to Cr(III) owing to the lower value of the reduction potential of Cr(III) to Cr^6+^. In addition, Cr^6+^ reduction to Cr(III) causes proton reduction in solution leading to its uptake by activated TETA-PP. Cr(III) ions remain in the water-soluble solution or form complexes with Cr-bonding groups which are discovered on the biosorbent surface (Hlungwane et al. 2018; Pertile et al. 2021).

To assess the least amount needed for optimum sorption, the optimization of the sorbent dosage is important (Aigbe et al. 2018b). Fig. 6c shows the impact of the sorbent dosage on the sequestration of Cr^6+^ onto the biosorbent. As observed in Fig. 6c, there was enhanced removal of Cr^6+^ ions as the dosage of sorbent employed in this study was increased from 1 to 3 g/L. This improved removal % with an increased dosage of sorbent used was attributed to supplementary active sorption sites that were available for the sorption of Cr^6+^ and was directly equivalent to the sorbent dosage. With the utilization of a low sorbent dosage, the amount of Cr^6+^ ions removed to the activated TETA-PP was noticed to be minimal owing to the inadequate sorption sites available for the sorbate molecules to reside (Aigbe et al. 2018a). The subsequent increase in the dosage of activated TETA-PP used led to increased removal efficiency of Cr^6+^ ions to the biosorbent, and this was ascribed to improved sorption sites on the surface of the biosorbent and as a further intensification in the high amount of dosage used led to a constant confiscation %. This constant rate of removal at high sorbent dosage was due to the *p* volume and surface saturation (Aigbe et al. 2020). At elevated biosorbent dosage, the creation of large blocks of sorbent particles led to the reduced available surface area for Cr^6+^ ion sorption (Al-Homaidan et al. 2018a).

In the sorption process, the *C*_o_ of the metal ions plays a critical role (El-Nemr et al. 2021). The preliminary concentration offers a critical mainspring necessary to overwhelm the mass transport challenge of metal ions between aqueous and solid forms (Konicki et al. 2018). The impact of the initial metal concentrations on the removal efficiency was investigated in this study (Fig. 6d), using initial metal concentrations of 100–400 mg/L in the presence of varying dosages of biosorbent (1–3 g/L), pH 2, and conducted at room temperature. The removal % of Cr^6+^ ions confiscated onto the biosorbent is noticed in Fig. 6d. The removal percentage of Cr6+ ion decreased from 99 to 81%, 100–94%, 100–98% and 100–99% with increasing Cr6+ concentration using a biosorbent dosage of 1, 1.5, 2.0, and 2.5 g/L, respectively. At a dosage of 3 g/L, the % of Cr^6+^ ion eliminated was constant at all concentrations of Cr^6+^ ions. The sorption efficiency was observed to depend on the reduced concentrations of Cr^6+^ ions that offer a definite force and improve the sorption process and the huge number of Cr^6+^ ions that resulted in the contest for the accessible binding sites in the biosorbent and hence enabled higher biosorption. Intensification in the concentrations of Cr^6+^ ions led to a slow decrease in the % of Cr^6+^ ions confiscated or additional Cr^6+^ ions left unabsorbed in the solution, and this was attributed to accessible binding site saturation (Al-Homaidan et al. 2018b).

*C*_*t*_ plays a very critical part in the dynamics of the sorption process, and knowledge about the kinetic sorption process assists to provide data on the sorption system design and cost (Radhika et al. 2019; Lima et al. 2021). *C*_*t*_ impact on the sequestration of Cr^6+^ ions to the synthesized activated TETA-PP is shown in Fig. 6e. The removal of Cr^6+^ ions to the biosorbent was observed to rapidly improve with the sorption process time of contact of 15 min and with EQB being attained in 30 min. The rapid removal of Cr^6+^ was ascribed to the huge active binding sites available and the extreme concentration gradient of the metal ions which improves the interaction between Cr^6+^ ions and the activated TETA-PP, while the filling of all active sites or *p* on the biosorbent with Cr^6+^ leads to the unavailability of sites for the further sorption of Cr^6+^ ions and thereby leads to the sorption process being at EQB (Shaikh et al. 2021).

### Kinetic models

The biosorption behaviour of Cr^6+^ to activated TETA-PP was studied using four kinetic models, which include “the pseudo-first-order (PFO), pseudo-second-order (PSO), Elovich (ELH) and intra-particle diffusion (IPD)” (Table 2). The linearized equations of the four kinetic models were used to explain the possible mechanism of sorption involved in the removal of Cr^6+^ ions from water-soluble solution, the rate of sorption and the significant rate-controlling steps (Shaikh et al. 2021). The most basic established equation used to study the sorption rate based on the sorption capacity is the linear equation of the Lagergren first-order model (El-Nemr et al. 2020a, 2020b, 2020c). With this model, it is not suitable for the overall sorption time determination in most instances. The linearized form of this model is depicted in Eq. (5).

5 $$\log \left({q}_{\mathrm{e}}-{q}_t\right)=\log \left({q}_{\mathrm{e}}\right)+\frac{k_1}{2.303}t$$

**Table 2:** Kinetic parameters of adsorption of Cr^6+^ ions to the activated TETA-PP

Parameter			PFO			PSO			
Activated TETA-PP (g/L)	Cr^6+^ conc. (mg/L)	*q*_e_ (exp.)	*q*_e_ (calc.)	*k*_1_ × 10^3^	*R*^2^	*q*_e_ (calc.)	*k*_2_ × 10^3^	*h*	*R*^2^
1.0	100	99.45	39.500	13.588	0.974	100.00	0.92	9225	0.997
	150	147.33	39.537	5.527	*0.761*	131.58	1.68	29070	1.000
	200	188.70	56.403	3.224	0.576	156.25	1.50	36630	0.996
	250	229.76	70.648	5.067	0.976	200.00	0.79	31447	0.998
	300	246.34	65.268	3.685	0.989	212.77	1.18	53476	1.000
	400	326.56	78.886	3.455	0.926	285.71	1.03	84034	1.000
1.5	100	66.67	4.300	8.291	0.767	65.79	14.72	63694	1.000
	150	100.00	27.791	14.739	0.896	101.01	1.45	14793	0.999
	200	132.65	16.665	5.988	0.905	126.58	3.85	61728	1.000
	250	162.85	35.917	9.442	0.771	156.25	1.37	33445	0.999
	300	195.45	40.290	6.448	0.976	181.82	1.39	46083	1.000
	400	250.30	74.405	3.685	0.882	212.77	0.93	42017	0.997
2.0	100	50.00	1.863	5.067	0.429	49.02	50.75	121951	1.000
	150	75.00	8.350	16.582	0.745	75.76	4.92	28249	1.000
	200	100.00	12.151	9.673	0.887	98.04	4.03	38760	0.999
	250	125.00	20.768	5.988	0.768	117.65	3.23	44643	1.000
	300	149.61	32.689	10.594	0.989	147.06	1.32	28653	0.999
	400	196.86	35.678	4.836	0.952	181.82	1.72	56818	0.999
2.5	100	40.00	2.152	8.061	0.870	39.53	26.56	41494	1.000
	150	60.00	2.826	9.903	0.758	59.52	20.02	70922	1.000
	200	80.00	4.250	6.679	0.883	78.74	15.36	95238	1.000
	250	100.00	7.976	9.442	0.943	99.01	6.54	64103	1.000
	300	120.00	14.348	8.982	0.833	117.65	3.39	46948	0.999
	400	159.06	18.243	6.218	0.642	153.85	3.55	84034	1.000
3.0	100	33.33	1.166	7.600	0.620	33.00	43.93	47847	1.000
	150	50.00	2.288	11.976	0.873	49.75	22.83	56497	1.000
	200	66.67	2.958	12.206	0.946	66.67	17.18	76336	1.000
	250	83.33	5.757	13.818	0.967	83.33	8.28	57471	1.000
	300	100.00	5.313	10.594	0.949	99.01	9.72	95238	1.000
	400	133.33	17.811	8.751	0.955	129.87	2.84	47847	1.000

*q*_e_ and *k*_1_ depict the sorption capacity at EQB (mg/L) and rate constant (1/min), which is assessed through an experiment from gradient and intercept from the log(*q*_e_ − *q*_*t*_) against *t* graph (El Nemr et al. 2010; Ugraskan et al. 2021; Sizirici et al. 2018; Bayuo 2021). In the PSO, the sorbate-sorbent interactions during the sorption procedure are taken into consideration. In this model, it is expected that the solute sorption rate is relative to the sorbent existing sites and the rate of reaction is reliant on the number of solutes on the sorbent surface’ hence, the driving force (*q*_e_ − *q*_*t*_) is relative to the amount of the accessible active sites on the sorbent (Kajjumba et al. 2018). Equation (6) gives the linearized form of this model.

6 $$\frac{t}{q_t}=\frac{1}{q_e^2{k}_2}+\frac{t}{q_{\mathrm{e}}}$$

*K*_2_ in g/mg min describes the PSO rate constant, and this is determined from the point of intersection of the linear graph drawn between *t* and 1 = *q* (Sizirici et al. 2018).

To further understand the chemisorption nature of sorption, the ELH model which was established by Zeldowitsch is utilized. This model was initially created to define the kinetics of the chemisorption of gas to solids (Table 3). This model assists in the prediction of the system mass and diffusion of surface, as well as the activation and deactivation energy. It is assumed in this model that the solute sorption rate reduces exponentially as the number of solutes sorbed is improved. Also, this model is a kinetic theory–based model which presumes that the sorption sites are enhanced with sorption, suggesting multilayer sorption (El Nemr et al. 2010; Kajjumba et al. 2018; Ayawei et al. 2017). The linear form of this model is expressed as Eq. (7).

7 $${q}_t=\frac{1}{\beta}\ln \alpha \beta +\frac{1}{\beta}\ln t$$

**Table 3:** Kinetic parameters of adsorption of Cr^6+^ ions to activated TETA-PP

Sorbent dose (g/L)	Cr^6+^ conc. (mg/L)	ELH			IPD		
		*β*	*α*	*R*^2^	*K*_*dif*_	*C*	*R*^2^
1.0	100	0.084	2.03 × 10^2^	0.9801	3.213	56.017	0.9761
	150	0.088	7.55 × 10^3^	0.8889	2.861	96.524	0.7738
	200	0.105	5.26 × 10^5^	0.6686	2.477	122.980	0.6142
	250	0.073	1.23 × 10^5^	0.8977	3.819	148.630	0.9617
	300	0.085	3.57 × 10^6^	0.9731	3.164	170.450	0.9643
	400	0.075	1.05 × 10^8^	0.9392	3.604	235.600	0.9459
1.5	100	0.606	2.03 × 10^15^	0.8785	0.413	60.713	0.7511
	150	0.097	9.10 × 10^2^	0.9488	2.696	66.223	0.8854
	200	0.235	1.74 × 10^11^	0.9653	1.122	112.350	0.9144
	250	0.069	4.65 × 10^3^	0.8455	3.617	113.060	0.7205
	300	0.098	2.91 × 10^6^	0.9877	2.750	147.090	0.9836
	400	0.086	3.41 × 10^6^	0.8179	3.243	166.250	0.8799
2.0	100	1.415	9.24 × 10^27^	0.6281	0.167	47.261	0.4810
	150	0.179	3.21 × 10^4^	0.6822	1.338	60.125	0.5362
	200	0.282	1.68 × 10^10^	0.8863	0.941	85.762	0.8533
	250	0.159	5.13 × 10^6^	0.8626	1.577	98.043	0.7460
	300	0.105	1.83 × 10^5^	0.9443	2.614	111.750	0.9678
	400	0.147	9.83 × 10^9^	0.8916	1.905	155.950	0.9530
2.5	100	1.532	6.37 × 10^23^	0.9015	0.170	37.336	0.8319
	150	0.863	1.56 × 10^20^	0.8626	0.293	56.042	0.7526
	200	0.811	3.53 × 10^25^	0.9592	0.320	74.660	0.8847
	250	0.406	3.19 × 10^15^	0.9489	0.657	90.277	0.9258
	300	0.297	1.67 × 10^3^	0.8537	0.937	104.360	0.9057
	400	0.161	1.86 × 10^9^	0.7282	1.534	134.250	0.6051
3.0	100	5.079	2.89 × 10^69^	0.5121	0.059	32.135	0.6225
	150	1.173	1.19 × 10^23^	0.9001	0.221	47.092	0.8249
	200	1.109	4.35 × 10^29^	0.9635	0.243	63.294	0.9578
	250	0.629	3.31 × 10^20^	0.9421	0.439	77.286	0.9830
	300	0.628	9.60 × 10^24^	0.9544	0.4283	93.801	0.9458
	400	0.203	6.02 × 10^9^	0.9516	1.3383	112.35	0.9585

*α* and *β* depict the initial sorption rate (mg/g min) and the desorption constant (g/mg) (Bayuo 2021). The ELH optimum sorption capacity and the ELH constant were estimated from the gradient and intercept of the graph of ln(*q*_e_/*C*_e_) against *q*_e_ (Ayawei et al. 2017). To assess the rate-limiting step during sorption, IPD is extensively employed. The solute sorption in a solution includes the mass transfer of the sorbate (film diffusion), diffusion on a surface and diffusion via *p*. Surface and *p* diffusion may happen concurrently while film diffusion is an autonomous step. IPD is examined using the Weber and Morris model of 1963 (Kajjumba et al. 2018). Equation (8) expresses the linear form of this model.

8 $${q}_t={t}^{0.5}{k}_i+C$$

**Fig. 7:**
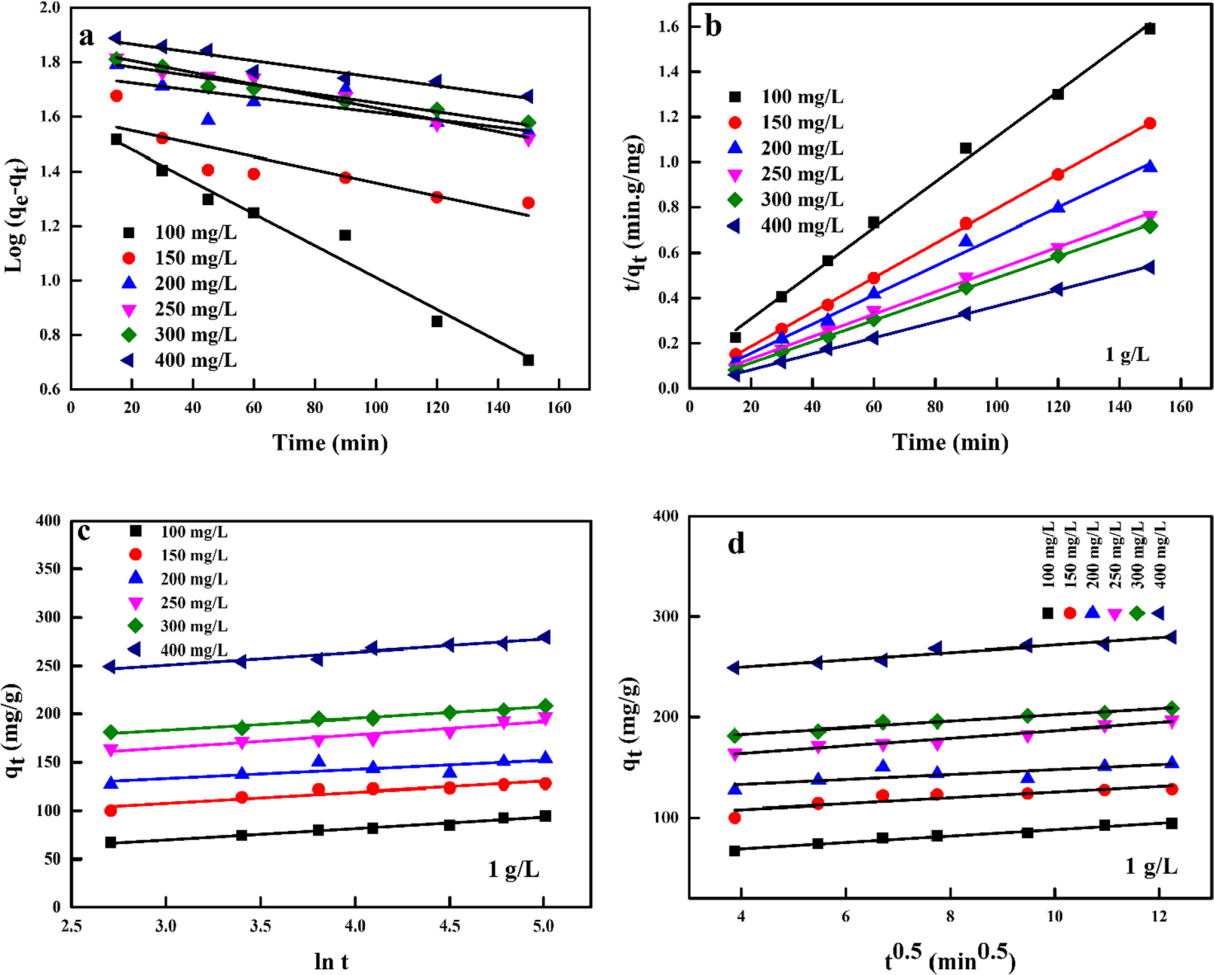
Graphs of **a** PFO, **b** PSO, **c** ELH and **d** IPD models

*K*_d_ is the IPD rate constant (mg/g min) and is determined from the graph of *q*_*t*_ against *t*^0.5^, and *C* indicates the intercept (Mangwandi et al. 2020; Bayuo 2021). From the linear plots of the four models employed for this study as shown in Fig. 7, it was observed that the PSO model gave a better coefficient of regression (*R*^2^), which was >0.9 for all computed values and which was close to unity when compared with those of other models employed (Table 2). Comparing the obtained *q*_e_ (calc.) results with the experimental data, very good conformity was observed (Şimşek et al. 2022).

Hence, the PSO model best defines the sorption of Cr^6+^ ions onto the biosorbent, and determined parameters from this model were close to the experimental data from the sorption process. Also, based on the best fitting of this model, chemisorption was a rate-limiting step of the sorption of Cr^6+^ ions to the biosorbent and this was also the findings from the studies (Bayuo 2021; Pertile et al. 2021; Ahmad et al. 2017; Zhao et al. 2021).

### Isotherm models

Imperatively, information on the sorption systems, the process of sorption, the sorbents’ surface properties and the association between the sorbates and sorbent is provided by isotherms (Aigbe et al. 2021; Marques et al. 2019). In this study, four models of isotherm, which include “Dubinin-Radushkevich (D-R), Freundlich (FRH), LNR and Temkin (TMN) models”, were employed to study the sorption EQB, favourability, mechanism and binary (liquid and solid) form distribution of the molecules of the sorbate and sorbent (Shaikh et al. 2021). The LNR isotherm is frequently utilized in liquid/solid structures to define the saturated monolayer sorption and to assess the sorption capacity for the sorbate concentration of a certain range; Eq. (9) was used for the linearized form of this model.

9 $$\frac{C_{\mathrm{e}}}{q_{\mathrm{e}}}=\frac{1}{q_{\mathrm{m}}}{C}_{\mathrm{e}}+\frac{1}{K{q}_{\mathrm{m}}}$$

*C*_e_, *q*_e_, *q*_m_ and *K* represent the EQB concentration of Cr^6+^ in milligrams per litre, the amount of Cr^6+^ ions sorbed to the biosorbent at equilibrium in milligrams per gram, the complete monolayer sorption capacity of the sorbent in milligrams per gram and the sorption EQB constant in litres per milligram. The LNR constants (*q*_m_ and *K*) are established from the gradient of the linear plot of *C*_e_/*q*_e_ against *C*_e_ (Aigbe et al. 2018a; Radhika et al. 2019), while *K* is employed to assess the dimensionless EQB parameters (*R*_L_) to determine if the sorption process is favourable or not. It is assessed employing Eq. (10) (Inyinbor et al. 2016).

10 $${R}_{\mathrm{L}}=\frac{1}{\left(1-{KC}_{\mathrm{o}}\right)}$$

In an experimental design, the FRH model is employed to explain sorption on an unstable surface (Aigbe et al. 2020; Gao et al. 2019). It is understood that sorption sites are distributed exponentially to sorption heat and the sorption rate varies on the energy strength of the sorption sites. The linearized expression of this model is given by Eq. (11) (Ukhurebor et al. 2021c; El Nemr et al. 2010).

11 $$\log {q}_{\mathrm{e}}=\frac{1}{n}\log {C}_{\mathrm{e}}+\log {K}_{\mathrm{f}}$$

**Fig. 8:**
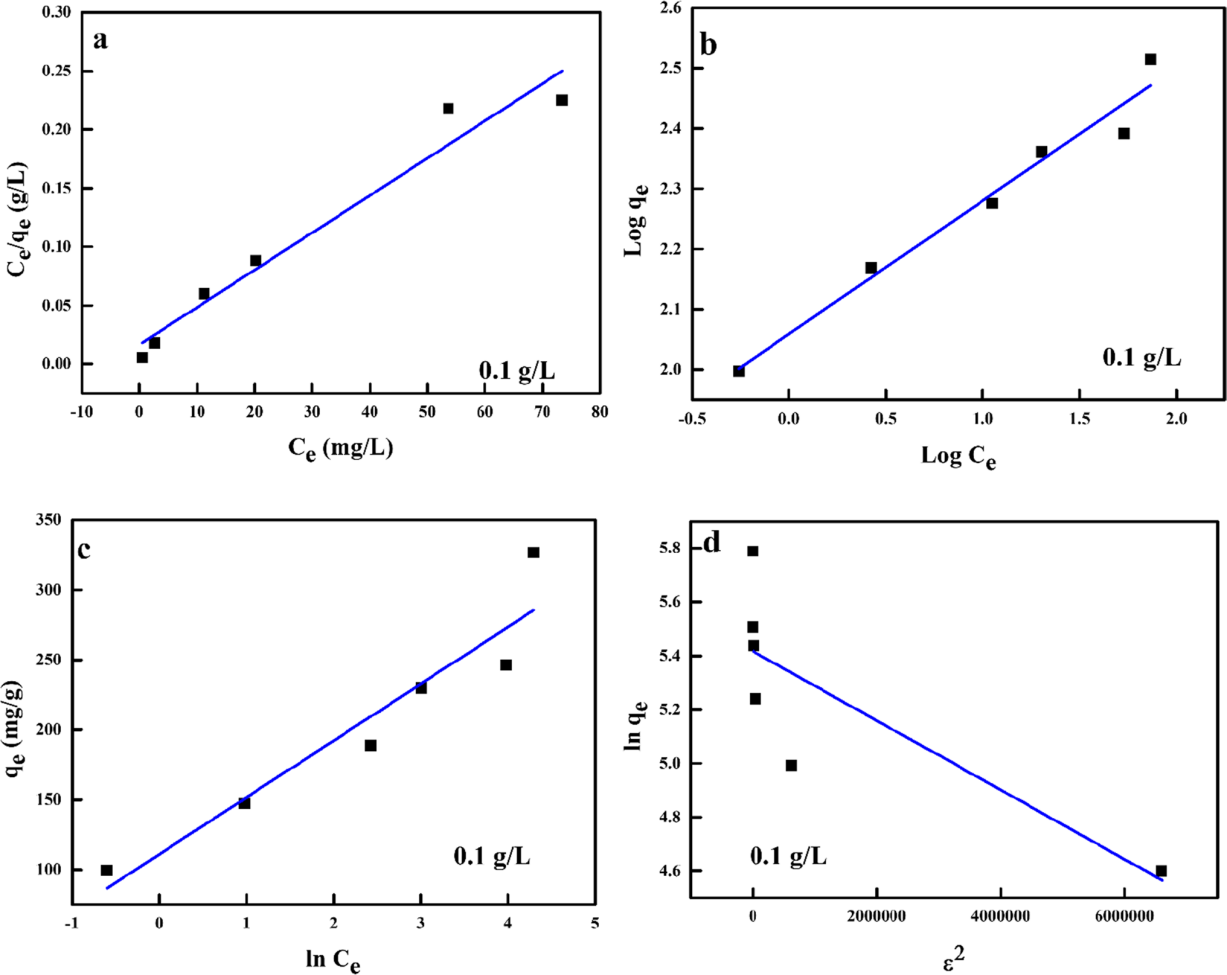
The plots of **a** LNR, **b** FRH, **c** TMN and **d** D-R isotherm models

*K*_f_ and *n* represent the FRH constants which include the parameters that impact the sorption capacity and sorption intensity respectively. Log *q*_e_ vs log *C*_e_ plots give an undeviating graph with the *K*_f_ and *n* values considered from the intercept and gradient (Inyinbor et al. 2016). The TMN model is used for investigating the sorption heat and the sorbate and sorbent interaction in a sorption process (El Nemr et al. 2010; Aigbe and Osibote 2021). According to this model, due to the sorbate-sorbent interactions, the molecules’ sorption heat in the layer reduces linearly with coverage due to the molecules’ sorption heat and the sorption is described by the unchanging distribution of the binding energies (Siddiqui et al. 2021). Equation (12) depicts the linearized form of this model.

12 $${q}_{\mathrm{e}}=\beta \ln A+\beta \ln {C}_{\mathrm{e}}$$

where $\beta =\frac{RT}{b}$ and *T*, *R* and *b* signify the total temperature in kelvin (K), universal gas constant (8.30 J/(mol K)) and the heat of sorption constant (El-Nemr et al. 2020a, 2020b, 2020c; El Nemr et al. 2010). To comprehensively understand the mechanism of the sorption processes whether they are physical or chemical processes and evaluate the superficial sorption energy, the D-R model is utilized. The linearized form of this model is represented by Eq. (13) (Aigbe and Osibote 2021):

13 $$\ln {q}_{\mathrm{e}}=\ln {q}_{\mathrm{m}}-K{\varepsilon}^2$$

where $\varepsilon = RT\ln \left(1+\frac{1}{C_{\mathrm{e}}}\right)$. *q*_m_, *ε* and *K* represent the theoretic sorption capacity (mg/g), the Polanyi potential and the sorption free energy per sorbate molecule constant (mol^2^/kJ^2^). The *K* value is derived from the gradient of the graph of ln *q*_e_ against *ε*^2^ (Ugraskan et al. 2021). Results of the experimental data fitting employing various isotherm models and their plots are shown in Fig. 8 and Table 4. From Table 4, it was noticed that the LNR isotherm well defined the sorption of Cr^6+^ ions to activated TETA-PP in this study with a coefficient of regression (*R*^2^) of >0.96 for biosorbent dosages used (1.0 g/L) when compared to other models used. This indicates that the sorption of Cr^6+^ ions to activated TETA-PP was well defined using this model and the maximum monolayer capacity gotten was 312.50 mg/g for activated TETA-PP biosorbent. This was suggestive of monolayer sorption of Cr^6+^ ions to activated TETA-PP biosorbent. The *R*_L_ for the sorption of Cr^6+^ ions to the biosorbent was assessed to be favourable sorption of Cr^6+^ ions to the activated TETA-PP biosorbent as both *K* and *C*_o_ have positive values (linear (*R*_L_ = 1), favourable (0 < *R*_L_ < 1), unfavourable (*R*_L_ > 1) and irreversible (*R*_L_ = 0) (Mutongo et al. 2014). An assessment of the maximum monolayer capacity is shown in Table 5. It is observed that the activated TETA-PP biosorbent showed better sorption of Cr^6+^ ions employing different biosorbents and could be applied in environmental redress practice.

**Table 4:** Isotherm parameters of adsorption of Cr^6+^ ions to activated TETA-PP

Isotherm model	Isotherm parameter	Activated TETA-PP
		1.0 g/L
LNR	*q*_m_	312.50
	*K*	0.19
	*R*^2^	0.96
FRH	*1*/*n*	0.22
	*K*_F_	114.63
	*R*^2^	0.970
TMN	*A*_T_	15.17
	*B*_T_	40.62
	*R*^2^	0.91
D-R	*q*_m_	225.29
	*K*	1.00 × 10^−7^
	*K* × 10^6^	0.10
	*E*	2236.07
	*R*^2^	0.66

**Table 5:** Comparison of the *q*_m_ of some biosorbents for Cr^6+^ ion adsorption from water

Biosorbents	*q*_m_ (mg/g)	References
Activated TETA-PP	312.50	This study
Orange peel	31.40	Pertile et al. (2021)
*Fomitopsis pinicola*	45.10	
Mixture of cones	41.00	
Peach stones	25.50	
Apricot stones	10.40	
Walnut shells	37.70	
Fleece	40.30	
Amine-modified passion fruit peel biosorbent	675.65	Zhao et al. (2021)
Powder of potato peelings	3.30	Mutongo et al. (2014)
*Moringa stenopetala* seed powder (MSSP)	9.71	Badessa et al. (2020)
Banana peel powder (BPP)	7.40	Badessa et al. (2020)
Nano-composites of the functionalized MWCNTs-quartzite nano-composite decorated with the stem bark extract of *Dacryodes edulis*	192.50	Amaku et al. (2021)
*Araucaria cunninghamii* Linn.	9.16	Dilshad et al. (2021)
Mango (M)	517.24	Giri et al. (2021)
Jackfruit (JF)	207.60	Giri et al. (2021)

### Mechanism of adsorption

The mechanism of Cr^6+^ ion biosorption to activated TETA-PP can be clarified based on four phases, which include sorption coupled with reduction, anionic sorption, reduction and cationic sorption and anionic and cationic sorption (Fan et al. 2017). As a result of the obtained biosorption experimental data and activated TETA-PP characterization results, the confiscation of Cr^6+^ ions to the biosorbent consists of sorption combined with reduction.

From the FTIR study, the presence of –OH, C=O, C–O and –NH_2_ was involved in the sorption combined with the reduction process. The sorption mechanism suggests that the first three phases for the sorption of Cr^6+^ ions are from the bulk stage to the solid stage; hence, the first phase of the rapid sorption of Cr^6+^ ions to the functional active sites on the biosorbent surface was by electrostatic attraction following the surface protonation of the sites on the activated TETA-PP biosorbent (Rzig et al. 2021). Also, Cr^6+^ ion removal by activated TETA-PP biosorbent is enabled by the surface complexation due to the carboxyl FG’s presence and the sorbed Cr^6+^ ions to the biosorbent maintained a varied redox reaction to create Cr^3+^ ions as signified by Eq. (14).

14 $${{\mathrm{H}\mathrm{CrO}}_4}^{-}+7{\mathrm{H}}^{+}+3{e}^{-}\rightleftharpoons {\mathrm{Cr}}^{3+}+4{\mathrm{H}}_2\mathrm{O}$$

**Fig. 9:**
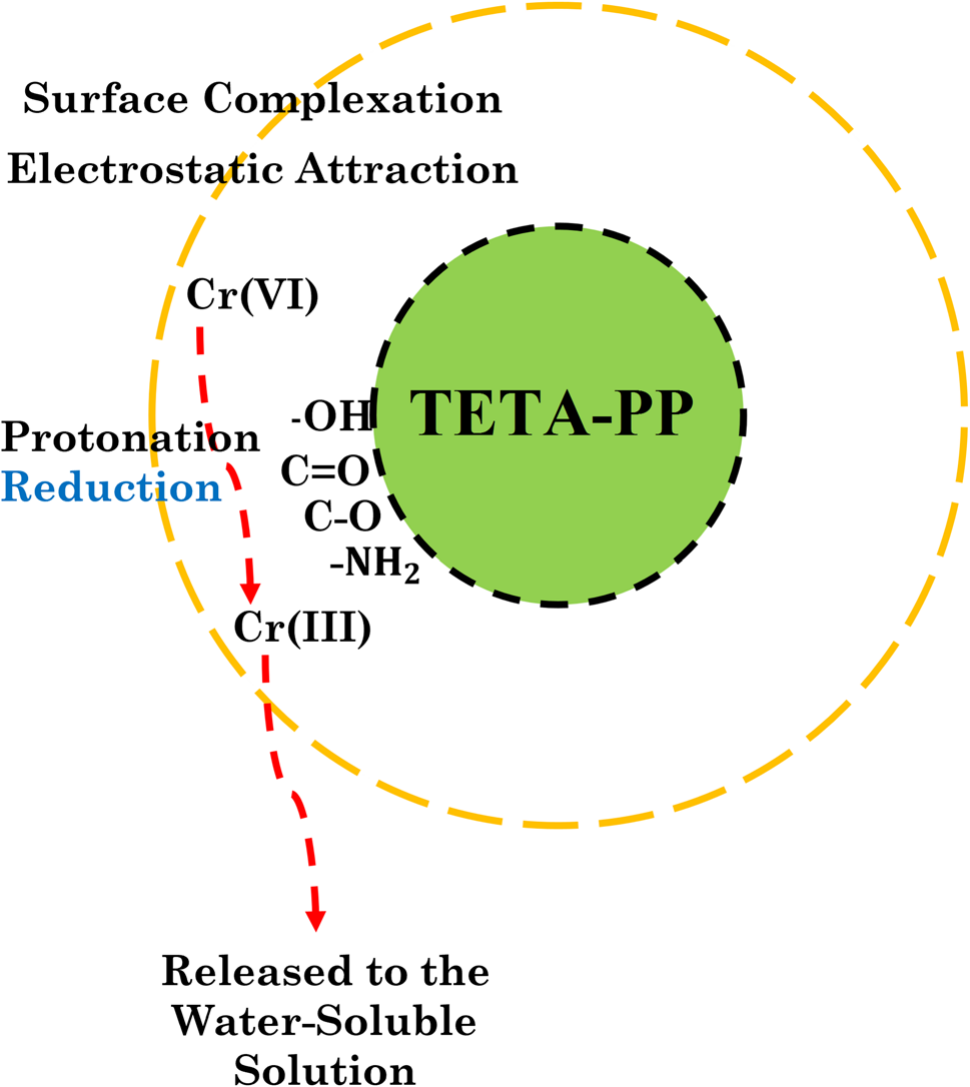
Intended mechanism of Cr^6+^ ion adsorption to activated TETA-PP biosorbent

The Cr^6+^ ion was reduced to a Cr^3+^ ion in a strongly acidic medium, which provides excessive H^+^ sites for Cr reduction. The reduced Cr^3+^ ion was efficiently engrossed to the functional active sites via electrostatic attraction and surface complexation. The affinity of active anionic sites on the activated TETA-PP biosorbent surface for Cr^3+^ ions enhanced the attaching of Cr ions to the biosorbent (Fan et al. 2017; Rzig et al. 2021). Fig. 9 shows the anticipated mechanism of Cr^6+^ ion sorption to activated TETA-PP biosorbent

### Regeneration of activated TETA-PP

**Fig. 10:**
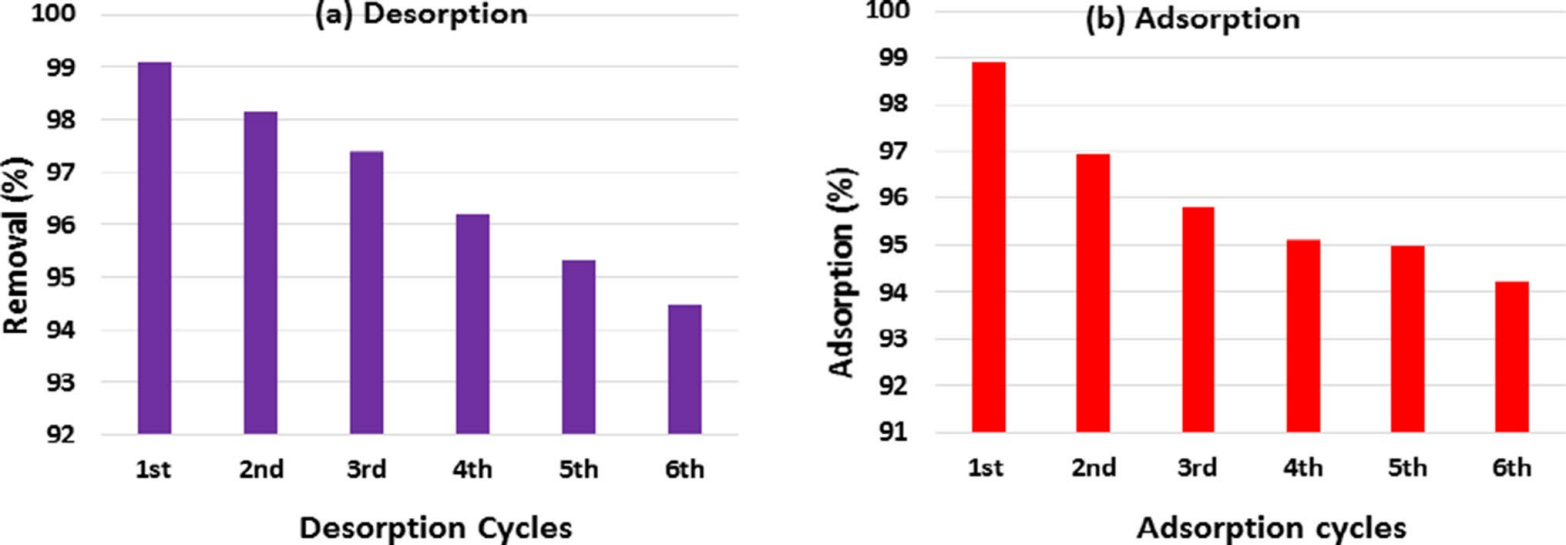
The effect of the regeneration rounds (cycles): **a** desorption percentage of Cr^6+^ ions from activated TETA-PP and **b** adsorption % of Cr^6+^ ions on activated TETA-PP using 3.0 g/L adsorbent dose and 4.0 g/L, *C*_0_ of Cr^6+^ ion at 25 ± 2 °C

Regeneration is an extensively used technique for the adsorption of effluents from water that relies critically on the reuse or recycling of adsorbent constituents, and the economics of this technique is highly dependent on the reuse or recycling of these adsorbent constituents as well. The chemical regeneration process was utilized in regenerating the depleted activated TETA-PP. The activated TETA-PP was regenerated (recycled) by washing it with 0.1 M NaOH solution, 0.1 M HCl solution and then pure water under high temperature, followed by drying it. It was noticed that the maximum removal of Cr^6+^ ions by activated TETA-PP was 94.23% after six regeneration rounds (cycles), respectively (Fig. 10), indicating that activated TETA-PP can regenerate.

### Optimization study

The optimization study was carried out to find the best pre-treatment process for the activated TETA-PP that has the maximum *q*_*t*_ (Singh and Ghatak 2020). The design matrix was used to explore the interaction impacts of three essential parameters, including contact time, biosorbent dose and initial Cr^6+^ ion concentration, on the removal of Cr^6+^ ions. Contact time, biosorbent dose and initial Cr^6+^ ion concentration were all investigated. Table 6 depicts the experimental design as well as the responses obtained. Based on the obtained results, the following polynomial equations (Eqs. (15) and (16)) for the removal of Cr^6+^ ions were constructed:

15 $$\mathrm{Removal}\%\mathrm{for}\ \mathrm{Coded}\ \mathrm{Factors}=88.33+9.51A-5.96B+7.33C+2.44 AB-2.40 AC+0.2071 BC$$

16 $$\mathrm{Removal}\%\mathrm{for}\ \mathrm{Actual}\ \mathrm{Factors}=73.44153+8.27859A-0.073882B+0.142833C+0.016261 AB-0.029056 AC+0.000017 BC$$

**Table 6:** Experimental design for adsorption of Cr^6+^ ions on activated TETA-PP

Run	Independent factors			Response %	
	*A*, dosage (g/L)	*B*, initial concentration (mg/L)	*C*, time (min)	Exp.	Predicted
1	3	100	15	97.33	96.63
2	1	250	90	73.01	77.93
3	3	400	180	100.00	99.46
4	3	200	120	98.63	100.34
5	1.5	400	120	73.78	78.78
6	1	100	60	81.72	82.89
7	3	400	180	100.00	99.46
8	3	100	15	97.33	96.63
9	3	200	120	98.63	100.34
10	1	400	15	62.32	60.48
11	2	250	15	79.41	80.99
12	3	400	15	88.09	89.17
13	1	100	150	94.35	93.28
14	3	400	15	88.09	89.17
15	2	250	150	92.72	92.99
16	2	100	90	97.72	93.64
17	1	400	15	62.32	60.48
18	2.5	400	90	93.02	87.77
19	2	100	180	100.00	101.41
20	1	250	180	91.90	88.55

Making predictions about the reaction for different values of each factor using the equation expressed in terms of actual factors is possible. It is necessary to specify the levels in the original units for each factor in this section. To evaluate the relative importance of each element, this equation should not be employed because the coefficients are scaled to fit the units of each factor and the intercept is not located in the centre of the design space.

**Fig. 11:**
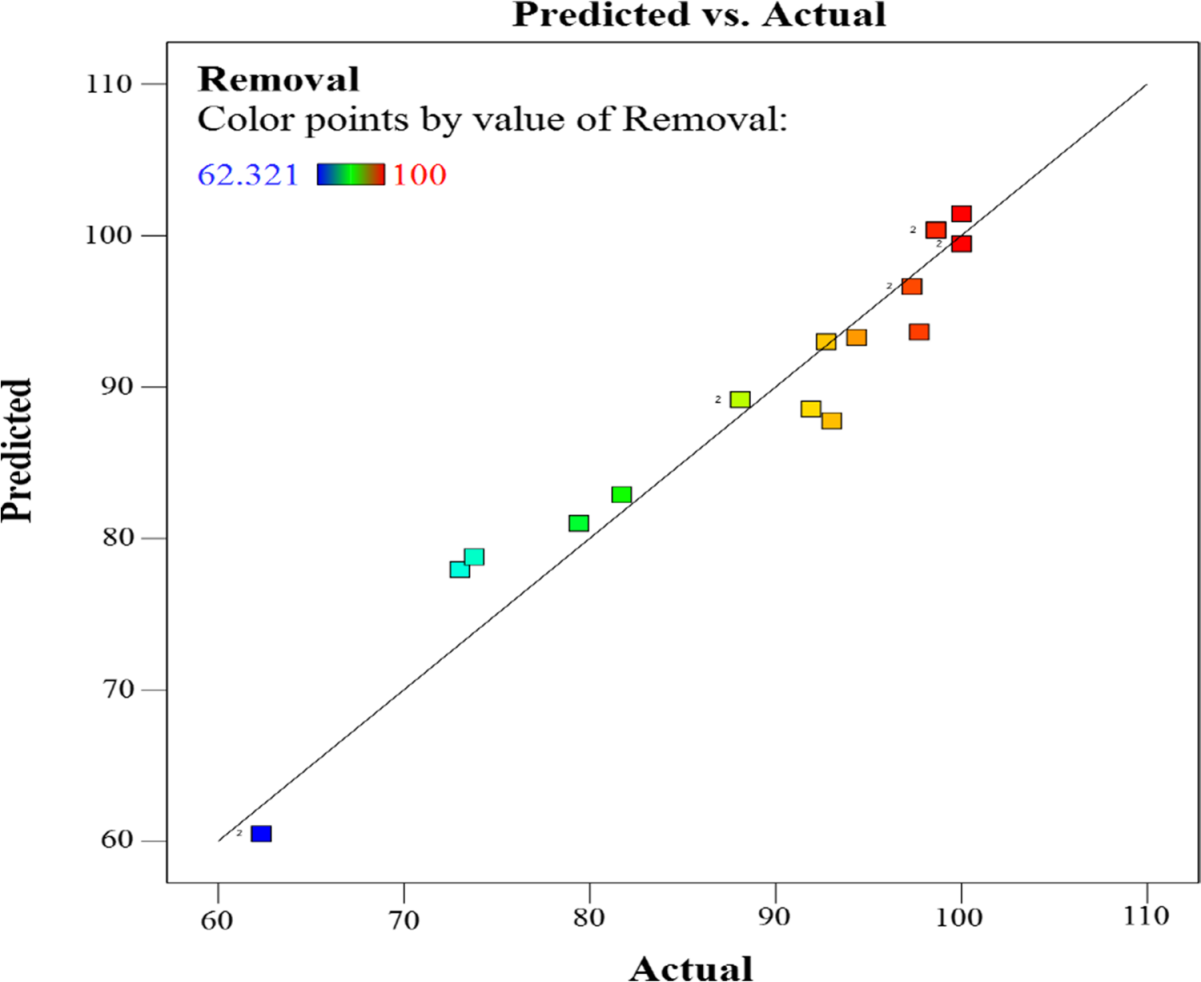
Plots between the predicted and experimental data for Cr^6+^ ion adsorption

According to Fig. 11, there is a positive association between anticipated and actual adsorption (percentage) of Cr^6+^ ions on TETA-PP. There appears to be good agreement between the experimental results and the projected model, which is supported by the high value of the correlation coefficient (*R*^2^ = 0.9607), as shown in the figure. This study employed an ANOVA to predict the cubic, individual and interaction effects of the independent factors on the adsorption of Cr^6+^ ions on TETA-PP. The results of the study are shown in Table 7. According to the findings, the quadratic model (with a *p*-value less than 0.05) makes a substantial contribution. *R*^2^ values show a good correlation between the predicted and exponential data (Table 7) (Amirov and Vakhshouri 2020; Isam et al. 2019; Sawood et al. 2021; Dil et al. 2019). The polynomial model’s standard deviation was described by the determination coefficient as a basis for the extent of deviation through the mean elucidated by the model, and the values of Adj-*R*^2^ and *R*^2^ show a good correlation between the predicted and exponential data (Amirov and Vakhshouri 2020; Isam et al. 2019; Sawood et al. 2021; Dil et al. 2019). The ANOVA results proved that the interaction between contact time (*A*) and the amount of the catalyst (*B*) on Cr^6+^ ion removal was significant with a *p*-value of 0.0428 (*p* value < 0.05). On the other hand, the interaction between the amount of the catalyst (*B*) and the initial concentrations (*C*) of Cr^6+^ ions was insignificant with a *p*-value of 0.0614 (*p* value < 0.05), where by raising the initial ion concentration, the removal % could be lowered.

**Table 7:** ANOVA and model fit summary for D-optimal design

Source	Value	Sum of squares	*df*	Mean square	*F*-value	*p*-value	Remarks	Source	*SD*	*R*^2^	Adjusted *R*^2^	Predicted *R*^2^	PRESS	Remarks
**Model**	-	2754.89	6	459.15	46.49	< 0.0001	Significant	-	-	-	-	-	-	-
*A*, time	-	990.21	1	990.21	100.27	< 0.0001	-	-	-	-	-	-	-	-
*B*, dose	-	365.94	1	365.94	37.05	< 0.0001	-	-	-	-	-	-	-	-
*C*, conc.	-	483.05	1	483.05	48.91	< 0.0001	-	-	-	-	-	-	-	-
*AB*	-	49.78	1	49.78	5.04	0.0428	-	-	-	-	-	-	-	-
*AC*	-	41.38	1	41.38	4.19	0.0614	-	-	-	-	-	-	-	-
*BC*	-	0.3084	1	0.3084	0.0312	0.8625	-	-	-	-	-	-	-	-
**Residual**	-	128.39	13	9.88	-	-	-	-	-	-	-	-	-	-
Lack of fit	-	128.39	8	16.05	-	-	-	-	-	-	-	-	-	-
Pure error	-	0.0000	5	0.0000	-	-	-	-	-	-	-	-	-	-
**Cor. total**	-	2883.28	19	-	-	-	-	-	-	-	-	-	-	-
***SD***	3.14	-	-	-	-	-	-	-	-	-	-	-	-	-
**Mean**	88.52	-	-	-	-	-	-	-	-	-	-	-	-	-
***CV*%**	3.55	-	-	-	-	-	-	-	-	-	-	-	-	-
***R***^**2**^	0.9555	-	-	-	-	-	-	-	-	-	-	-	-	-
**Adjusted** ***R***^**2**^	0.9349	-	-	-	-	-	-	-	-	-	-	-	-	-
**Predicted** ***R***^**2**^	0.9064	-	-	-	-	-	-	-	-	-	-	-	-	-
**Adeq. precision**	22.0176	-	-	-	-	-	-	-	-	-	-	-	-	-
Linear	-	-	-	-	-	-	-	-	3.87	0.9169	0.9014	0.8778	352.44	
2FI	-	-	-	-	-	-	-	-	3.14	0.9555	0.9349	0.9064	269.77	Suggested
Quadratic	-	-	-	-	-	-	-	-	3.37	0.9607	0.9253	0.7760	645.81	
Cubic	-	-	-	-	-	-	-	-	8.143E−06	1.0000	1.0000		*	Aliased

*R*^2^ is a basic matrix which tells you about how much variance is explained by the model. The *R*^2^ value will always increase irrespective of the variable significance. The adjusted R^2^ is the calculated R^2^ from only those variables whose addition in the model which are significant. *R*^2^ explains the degree to which your input variables explain the variation of your output/predicted *R*^2^ variable. So, in simple terms, the higher the *R* squared, the more variation is explained by your input variables, and hence, the better is your model. Typically, the more non-significant variables you add into the model, the gap in *R*^2^ and adjusted *R*^2^ increases

According to the predicted *R*^2^ of 0.7760, the adjusted *R*^2^ of 0.9253 is in reasonable agreement with the predicted *R*^2^ of 0.9253; in other words, the difference is less than 0.2. Adeq. precision is a signal-to-noise ratio (SNR) measurement tool. It is preferable to have a ratio bigger than 4. A signal-to-noise ratio of 22.0176 suggests a sufficient signal, which demonstrates a strong RSM model signal that can be used for navigation of the design (Sawood et al. 2021; Bagheri et al. 2019).

### Simultaneous adsorption variables’ effects

**Fig. 12:**
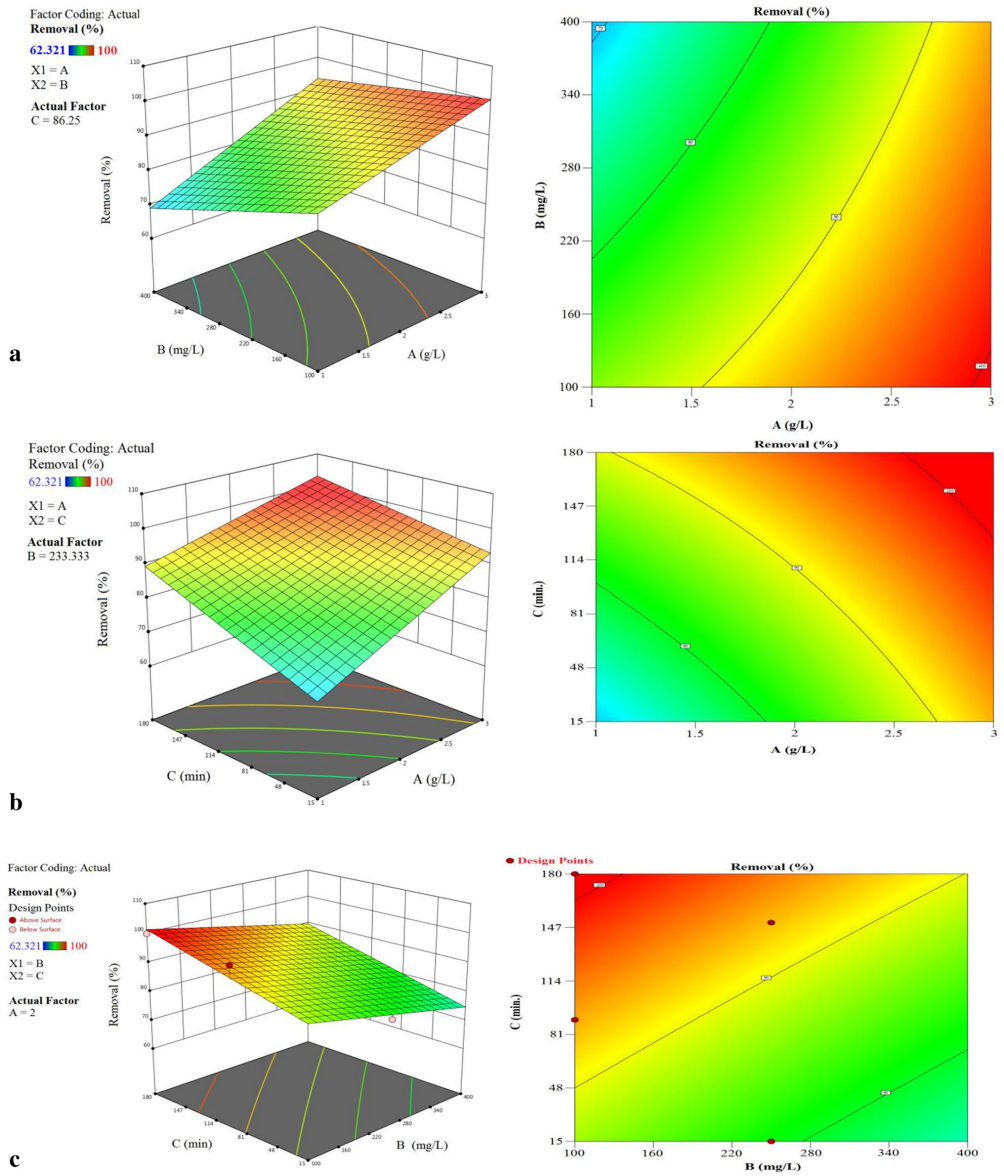
The combined effect of process variables **a** initial concentration and adsorbent, **b** adsorbent dosage and contact time and **c** initial concentration and contact time on Cr^6+^ ion removal % with interaction effect of dual factors

Figure 12 shows three-dimensional surface plots that depict the impacts and interactions of independent variables, namely the adsorbent dose, the initial Cr^6+^ ion concentration and the contact time, on the removal percentages of Cr^6+^ ion as responses.

**Fig. 13:**
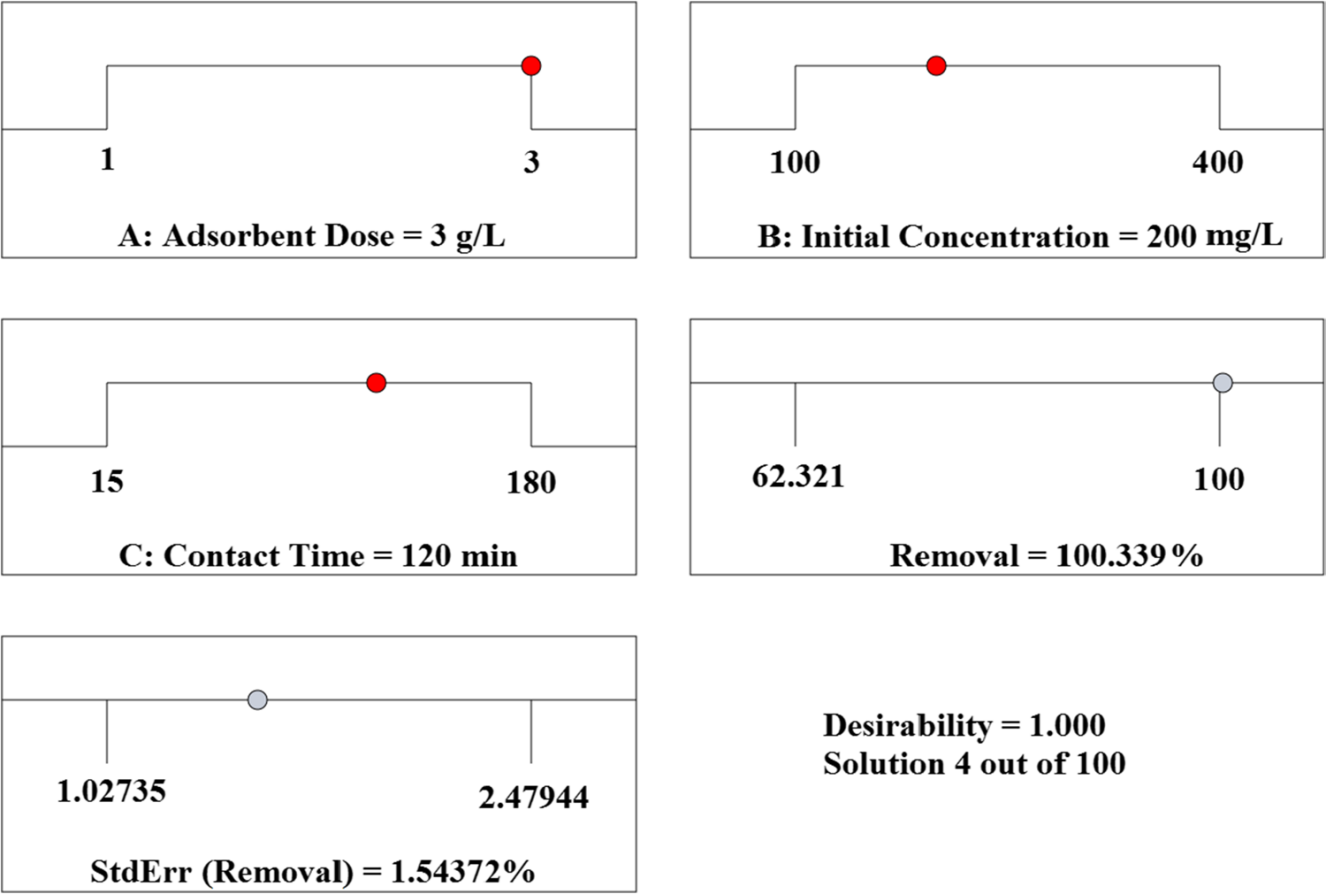
Optimum conditions predicted by the RSM method

The relationship between the starting ion concentration and the adsorbent dosage, as shown in Fig. 12a, reveals that both parameters have a considerable impact on the removal of Cr^6+^ ions from the solution. The clearance percentages rose as the amount of adsorbent used increased. Due to the existence of additional active sites as well as a large adsorbent surface area that is readily available for adsorption (Isam et al. 2019; Mondal et al. 2019), this result was achieved. By raising the initial ion concentration, the removal % could be lowered (Fig. 12b and c). The limited number of active sites on the adsorbent surface observed at high adsorbate concentrations (Roy et al. 2017) could explain this observation. By increasing the residence duration from 30 to 90 min, it was possible to enhance the removal %. These findings confirmed that the initial adsorption rate was extremely quick, owing to the availability of a high surface area and the abundance of unused sites on the adsorbent surface (Lingamdinne et al. 2018), as previously reported. The difficulties in reaching the remaining unoccupied locations may be contributing to the slowdown in ion removal. Figure 13 illustrates how a complementary statistical design calculation was carried out under the same experimental conditions to optimize and validate the predicted mathematical model; this calculation revealed that the higher desirability value was equal to 1, which was obtained from the results of the mathematical model. Using these settings, the highest removal percent (removal % = 100) obtained corresponds to the following contact duration, biosorbent dose and concentration: 120 min, 3 g/L biosorbents and 200 mg/L concentration.

## Conclusion and recommendations

This study explores the utilization of biosorbents decorated by TETA prepared from PP as biosorbents (activated TETA-PP) for the sequestration of Cr^6+^ ions from water bodies. Some factors, such as pH, adsorbent dosage, initial Cr^6+^ ion concentration and *C*_*t*_, were observed to influence the dynamic sequestration of Cr^6+^ ions employing the synthesized activated TETA-PP as a biosorbent. The sorption of Cr^6+^ ions to the activated TETA-PP biosorbent was well defined using the LNR isotherm and PSO models with an *R*^2^ of 0.96, and the estimated sorption capacity was 312.50 mg/g. In addition, the study shows that the activated TETA-PP biosorbent can conceivably have up to six regeneration or reusability rounds (circles). The results from this study have further confirmed the possibility and capability of utilizing cheap and sustainable biosorbents from agro-waste materials (such as the activated TETA-PP) for the confiscation of Cr^6+^ ions. It is therefore recommended that there should be continuous research for novel, cheaper and more sustainable biosorbents, especially from agro-waste materials that can be explored and utilized for the confiscation of HMs and other toxic chemicals from the environment. Also, future research should concentrate on using intelligent procedures to advance a general and precise correlation for the removal of HMs and other industrial toxic chemicals from the environment.

## Data Availability

Data sharing does not apply to this article.
